# Arginine dependency in omental metastasis of epithelial ovarian cancer reveals a therapeutic vulnerability

**DOI:** 10.1038/s41419-026-08606-3

**Published:** 2026-03-24

**Authors:** Jiming Tian, Ting Lei, Yulu Du, Dalin Wang, Haiping Liang, Zeyu Yan, Jun Xu, Wenhao Xue, Shuangbin Wang, Xuehan Bi, Dongdong Wang, Junfen Li, Yucun Wang, Xiaolei Liang, Yongxiu Yang

**Affiliations:** 1 The First Clinical Medical College of Lanzhou University, Lanzhou, 730000 Gansu China; 2 Department of Obstetrics and Gynecology, the First Hospital of Lanzhou University, Gansu Provincial Clinical Research Center for Gynecological Oncology, Lanzhou, 730000 Gansu China; 3 NHC Key Laboratory of Molecular Probes and Targeted Diagnosis and Therapy, The Fourth Hospital of Harbin Medical University, Harbin, 150000 Heilongjiang China; 4 Department of Hepatobiliary Surgery, Xijing Hospital, Fourth Military Medical University, Xi’an, 710000 Shaanxi China; 5 Department of Physiology and Pathophysiology, Fourth Military Medical University, Xi’an, 710000 Shaanxi China; 6 Department of Vascular Surgery, The First Hospital of Lanzhou University, Lanzhou, 730000 Gansu China; 7 Medical College of Yan’an University, Yan’an, 716000 Shaanxi China; 8 Department of Pathology, Lanzhou University Second Hospital, Lanzhou, 730000 Gansu China; 9 Gansu Provincial Cancer Hospital, Lanzhou, 730000 Gansu China

**Keywords:** Cancer metabolism, Mechanisms of disease

## Abstract

Epithelial ovarian cancer (EOC) is the leading cause of death among gynecological malignancies, and the tumors with advanced-stage are frequently characterized by extensive metastasis. Although metabolic reprogramming of amino acids represents a hallmark of cancer, its specific role in the metastatic progression of EOC remains poorly understood. Here, we identified a critical metabolic vulnerability in omental metastasis of EOC. Despite defective endogenous synthesis, arginine accumulation depends on exogenous uptake. In vivo experiments demonstrated that dietary arginine deprivation suppressed tumor growth and metastasis, whereas supplementation or enhanced uptake of arginine promoted tumor cell proliferation, invasion, and migration in vitro. Mechanistically, increased arginine binds to the RNA helicase DDX3X, inducing nuclear retention of DDX3X and further promoting the transcription of DNA damage response (DDR)-related genes, thereby facilitating DDR through activating the ATM/CHK2/P53 axis to enable cancer cells to survive under metastatic stress. Notably, arginine restriction or pharmacological inhibition of DDX3X did effectively suppress both primary tumor growth and omental metastasis in mouse models. Collectively, our findings reveal that arginine is a metabolic vulnerability in omental metastasis of EOC, indicating that arginine restriction and DDX3X inhibition represent promising therapeutic strategies.

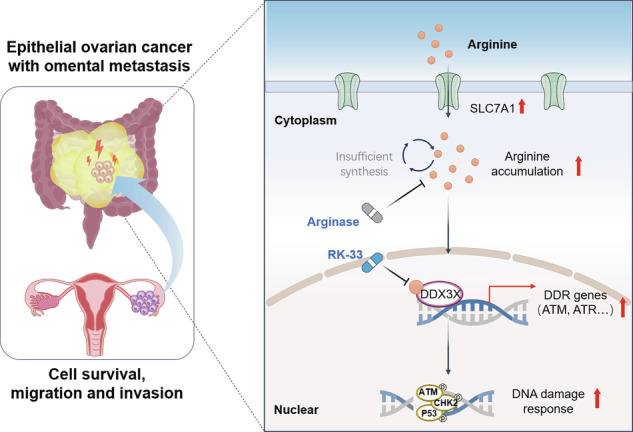

## Introduction

Epithelial ovarian cancer (EOC) is a highly fatal gynecological pelvic malignancy. In 2022, ~325,000 female patients were diagnosed with EOC, while almost 207,000 individuals died from the disease [1]. Furthermore, nearly 38,000 adolescent and young adult women are diagnosed with EOC, which severely impacts their health and fertility [2]. Although more than 70% of patients with early-stage EOC present with atypical symptoms, they are often ignored until diagnosed at advanced stages, accompanied by widespread peritoneal metastasis and malignant ascites that critically compromise surgical opportunities [3, 4]. The current treatment strategies for EOC include cytoreductive surgery and cytotoxic chemotherapy. However, the optimal timing for surgical intervention remains controversial, and the five-year survival rate of patients with EOC remains less than 50%. Considering the distinct biological characteristics between metastatic and primary tumors, the major challenge in EOC treatment lies in the limited understanding of metastatic mechanisms [5].

Recent studies have revealed that cancers are fundamentally metabolic diseases, with metabolic reprogramming recognized as a hallmark of malignancy [6]. During tumor progression and metastasis, metabolic phenotypes are significantly influenced by nutrient distribution and the metastatic microenvironment. Thus, tumor cells exhibit spatiotemporal heterogeneous metabolic characteristics via activating multi-dimensional adaptive mechanisms to adapt to the dynamic changes of the tumor microenvironment [7]. For example, unsaturated lipid metabolism plays a key role in the hematogenous metastasis of EOC, and metastatic EOC cells are more susceptible to ferroptosis inducers, along with higher lipid unsaturation [8]. Additionally, in response to genotoxic drug-induced stress, tyrosine decomposition is the main mechanism of fumarate production in EOC cells. Fumaric acid produced by fumarylacetoacetate hydrolase directly binds to REV1 DNA-directed polymerase (REV1), thereby inhibiting translesion DNA synthesis (TLS) and enhancing the sensitivity of EOC to genotoxic drugs [9]. A better understanding of the mechanisms of metabolic reprogramming and nutritional dependencies of EOC not only uncovers the metabolic vulnerabilities of tumor cells, but also provides a theoretical basis for developing precise therapeutic strategies targeting metabolic pathways.

It has been widely reported that amino acid metabolic reprogramming exerts an important influence on tumor survival, growth, metastasis, and drug resistance, and several studies have demonstrated that the non-classical functions of amino acids play crucial roles [10–12]. Hepatocellular carcinoma displays a universal metabolic property of urea cycle loss, which results in the impairment of arginine synthesis. Thus, the transporter solute carrier family 7 member 1 (SLC7A1), which supports the dependence on exogenous arginine, plays a vital role in sustaining the survival of HCC cells [13]. Additionally, high levels of arginine tend to drive the expression of metabolism-related genes by activating RNA-binding motif protein 39 (RBM39), thereby facilitating tumor growth [14]. Besides, impaired respiratory capacity limits cancer cell proliferation by restricting aspartic acid synthesis. To exploit this vulnerability, metformin inhibits tumor growth by depleting aspartate [15]. However, Aspartic acid also promotes collagen synthesis by activating N-methyl-D-aspartic acid receptor (NMDA) and inducing eukaryotic translation initiation factor 5 A (eIF5A) translation, thereby enhancing tumor cell invasion and metastasis [12]. Although amino acid metabolism has been extensively studied in primary tumors, its role in EOC metastatic progression was poorly characterized.

Here, we found a significant accumulation of arginine in the omental metastasis of EOC. We observed that arginine deprivation inhibited the growth and metastasis of EOC in vivo, while supplementation or enhanced uptake of arginine promoted the proliferation, migration, and invasion of cancer cells in vitro. Mechanistically, the accumulated arginine bound to the helicase DEAD-box helicase 3 X-linked (DDX3X) and induced its nuclear retention, which has been found overexpressed in primary EOC, yet little is known about its role in metastasis [16]. Furthermore, this interaction accelerated DNA damage response (DDR) by activating the ATM/CHK2/p53 axis. Moreover, a small-molecule inhibitor targeting DDX3X effectively suppressed tumor growth and metastasis. Collectively, these findings demonstrate that arginine restriction and targeting DDX3X are potential therapeutic strategies for EOC metastasis.

## Materials and methods

### Patient samples

Fresh-frozen tissues of primary tumors and paired omental metastatic foci from seven treatment-naive EOC patients (with TP53 wild‑type status confirmed by immunohistochemistry), along with formalin-fixed paraffin-embedded (FFPE) sections from an independent cohort of 20 EOC patients (Age [years], 59.3 ± 10.0; CA125 [U/mL], 1095.9 ± 1512.0; HE4 [pmol/L], 484.9 ± 412.5), were collected from the Department of Gynecology, First Hospital of Lanzhou University. In addition, peripheral blood samples were collected from five EOC patients with omental metastasis and five age-matched healthy individuals for the measurement of plasma arginine concentration. All diagnoses were confirmed pathologically. The present study was approved by the Ethics Committee of the First Hospital of Lanzhou University (LDYYLL2024-130). Written informed consent was obtained from all patients.

### Animal experiment

Female C57BL/6 mice (8 weeks old) were housed in a constant temperature, humidity-controlled, specific pathogen-free environment and allowed to eat and drink freely. The orthotopic ovarian cancer model was established according to Hertzog’s methods [17]. Anesthesia was induced and maintained under 2.5% isoflurane delivered via a nose cone. The surgical site was then depilated and prepared aseptically. A 1 cm incision was made over the left ovarian fat pad, and the ovary was gently exposed using a cotton swab. A total of 1 × 10⁶ ID8-LUC cells resuspended in 50 μL of ice-cold Matrigel were injected into the ovarian bursa using a 29G syringe. Finally, the peritoneum and the skin were sutured. Postoperatively, buprenorphine and antibiotics were administered to prevent infection. For the intraperitoneal (disseminated) ovarian cancer model, 5 × 10^6^ ID8-LUC cells in 200 μL of sterile PBS were injected directly into the abdominal cavity. After injection, mice were randomly assigned to receive either a control diet (Cat. No. M10005, Moldiets, Chengdu, China) or an arginine-deficient diet (Cat. No. M11712, Moldiets, Chengdu, China). One month later, D-Luciferin potassium salt (25 mg/kg, Cat. No. L10060, Psaitong Biotechnology, Beijing, China) was injected intraperitoneally, and bioluminescence imaging was performed 10 min post-injection using a small-animal in vivo imaging system (Caliper Life Science, Hopkinton, USA). At the experimental endpoint, mice were anesthetized and then euthanized by cervical dislocation. Ascites volume was measured, and tumor nodules from the abdominal cavity and the primary ovarian tumor were excised and weighed. Investigators were blinded to the group allocation throughout the experiment and during outcome assessment. Animal experiments were approved by the Institutional Animal Care and Use Committee of the First Hospital of Lanzhou University (LDYYLL2024-51).

### Cell lines and cell culture

The human epithelial ovarian cancer cell lines A2780 and HEY A8 were obtained from the American Type Culture Collection (ATCC). The murine ovarian cancer cell line ID8 stably expressing luciferase (ID8-Luc) was obtained from Shanghai Zeye Biotechnology (Cat. No. ZY-C6082M-L, Zeye Biotechnology, Shanghai, China). The human platinum-resistant ovarian cancer cell line A2780/CDDP (Cat. No. IMD-022, IMMOCELL, Xiamen, China), murine cell lines Hepa1-6 (hepatoma), MC38 (colon carcinoma), and C1498 (leukemia) was obtained from IMMOCELL. A2780 and Hey A8 cells were maintained in L-arginine-free RPMI 1640 medium (Cat. No. LA10015, BAIAOLAIBO, Beijing, China) supplemented with 10% dialyzed fetal bovine serum (FBS), 1% penicillin/streptomycin, and L-arginine at specified concentrations (e.g., 0, 100, and 500 μM). ID8-Luc, Hepa1-6, MC38, and C1498 cells were cultured in high-glucose DMEM (Cat. No. PM150210, PROCELL, Wuhan, China) supplemented with 10% FBS and 1% penicillin/streptomycin. Cell lines authentication was performed using short tandem repeat profiling, and routine mycoplasma contamination screening confirmed negative results.

### Plasmid construction and transfection

The SLC7A1 overexpression plasmid was synthesized and sequenced by WZ Biosciences (Cat. No. CH846661, WZ Biosciences Inc., Jinan, China). FLAG-tagged full-length DDX3X and its truncation mutants, and point mutants (N159A, T201A, R202A, and G227A) were cloned into the pcDNA™ 3.1 (+) vector. The truncation variants included: ΔN-IDR, deletion of amino acids 1–168 (intrinsically disordered N-terminal region); ΔC-IDR, deletion of amino acids 580–662 (C-terminal disordered domain); ΔHelicase, deletion of amino acids 169-579 (helicase core); ΔNES, deletion of amino acids 1–22 (nuclear export signal); and Δ23–168, deletion of amino acids 23–168 (partial N-terminal truncation). Small-interfering RNAs targeting human SLC7A1 (siSLC7A1) and DDX3X (siDDX3X), along with non-targeting scrambled controls, were designed and synthesized by Sangon Biotech (Shanghai, China). The siRNA sequences are listed in Supplementary Table 1. For stable knockdown, lentiviral particles expressing DDX3X-targeting shRNA were produced by cloning the shRNA sequences into the pLent-U6 vector and packaging in HEK293T cells by WZ Biosciences. Transient transfection of plasmids or siRNAs was performed using Lipofectamine 2000 (cat. No. 11668-019, Invitrogen, Carlsbad, CA, USA) according to the manufacturer’s protocol.

### Cell viability assay

Cells were plated in 96-well plates at a density of 2000 cells/well in 100 μL medium and cultured for the specified durations (24, 48, 72, or 96 h) at 37 °C under 5% CO₂. To assess cell viability, 10 μL of CCK-8 reagent (Cat. No. C0005, TargetMol, Shanghai, China) was added to each well and incubated for 3 h. Absorbance was measured at 450 nm using a Bio-Tek SynergyLX microplate reader (Bio-Tek, Winooski, USA).

### Wound-healing assay

The wound-healing assay was performed following previous protocols [18]. Briefly, cells were seeded in six-well plates and allowed to reach 80% confluence. A wound was scratched in the monolayer using a sterile 200 μL pipette tip. The detached cells were removed by gentle washing with PBS, followed by incubation in serum-free medium to minimize proliferation. Wound closure was monitored under a microscope at 0 and 48 h post-scratch. The wound area was measured using ImageJ software (National Institutes of Health, Bethesda, Maryland, USA), and relative wound closure was calculated as follows: (Area_0 h_ - Area_48 h_)/Area_0 h_.

### Transwell migration and invasion assays

Cell migration and invasion assays were performed using 24-well transwell inserts with polycarbonate membranes (8.0-μm pore size). For the migration assay, ovarian cancer cells were seeded into the upper chamber (transwell insert). The lower chamber of the plate well was filled with complete medium containing 20% FBS as a chemoattractant. After 24 h of incubation, non-migrated cells remaining on the apical (upper) side of the membrane were gently removed using cotton swabs. The transwell inserts were then fixed and stained with 0.2% crystal violet to visualize the migrated cells adherent to the basal (lower) side of the membrane. For quantification, five randomly selected fields per membrane were photographed and counted under a microscope. For the invasion assay, the membranes of the transwell inserts were precoated with Matrigel to form an extracellular matrix barrier. After 48 h of incubation, the same steps of removing non-invaded cells, fixing, and staining were performed.

### Protein extraction, western blotting, qRT-PCR, hematoxylin and eosin, and IHC

Protein extraction and western blotting were performed as described in previous studies [19]. Briefly, the indicated treated cells were collected, lysed on ice for 30 min using radioimmunoprecipitation assay buffer (RIPA) lysate supplemented with phenyl methane sulfonyl fluoride (PMSF) and a complete protease/phosphatase inhibitor, then centrifuged at 12,000 × *g* for 30 min at 4 °C, and the supernatant was collected as the total cell protein. Using the nuclear and cytoplasmic proteins extraction kit (Cat. No. P0027, Beyotime, Shanghai, China), the cytoplasmic and nuclear proteins were obtained according to the manufacturer’s instructions, and the protein concentration was quantified using a Bicinchoninic Acid Assay (BCA) protein assay kit (Cat. No. AP12L025, LIFE-ILAB BIOTECH, Shanghai, China). About 20 μg protein was separated by 10% SDS/PAGE gel, transferred to a 0.22 or 0.45 μm PVDF membrane, blocked with quick blocking solution (Cat. No. AP0291L, AccuRef Scientific, Xi’an, China), and incubated overnight with the primary antibody. The following day, the membranes were incubated with horseradish peroxidase (HRP)-labeled secondary antibody for 1 h at room temperature. Finally, the enhanced chemiluminescence (ECL) reagent (Cat. No. AP0081S, AccuRef Scientific, Xi’an, China) was used to expose the strips.

Hematoxylin and eosin (H&E) and immunohistochemistry (IHC) staining were employed as previously described [20]. The antibodies used in this study were listed in Supplementary Table 2. RNA extraction and quantitative reverse transcription polymerase chain reaction (qRT-PCR) were also performed as previously described [20]. The primers used were listed in Supplementary Table 3.

### Subcutaneous tumor models

Eight-week-old female BALB/c nude mice were housed in standard pathogen-free conditions. A2780 cisplatin-resistant cells during the exponential growth phase were harvested and resuspended in a 1:1 mixture of PBS and Matrigel at a density of 10⁷ cells/100 µL. Cells in 100 μL suspension were subcutaneously injected into the right flank of each mouse (*n* = 5 mice per group). The tumor sizes were measured by a vernier caliper every 7 days, and the tumor volumes were recorded based on the formula (length × width^2^)/2. The nude mice were sacrificed one month after injection, and the subcutaneous tumors were resected and weighed.

### Cell apoptosis assay

The proportion of apoptotic cells were evaluated based on the Annexin V/PI staining method by flow cytometry. Operations were carried out according to the instructions of Annexin V-FITC/PI Apoptosis Kit (Cat. No. E-CK-A211, Elabscience, Wuhan, China). In brief, cells with different treatments were digested with EDTA-free trypsin, then the digestion was promptly stopped with complete medium containing serum. Cells were washed twice with cold PBS and resuspended in 1× Annexin V Binding Buffer. Then the harvested cells were stained by FITC-conjugated annexin V and PI for 10 min in the dark. Finally, 400 µL of 1× Binding Buffer was added to each sample, and cells were analyzed immediately using a flow cytometer (NovoCyte Quanteon, Agilent Technologies, Inc, Singapore).

### Preparation and protein enrichment of arginine-coupled NHS magnetic beads

L-Arginine was immobilized onto *N*-hydroxysuccinimide (NHS)-activated magnetic beads (Cat. No. 70703-5, Beaverbio, Suzhou, China) according to the manufacturer’s instructions. The NHS magnetic beads feature an 8-atom hydrophilic spacer arm. Arginine was dissolved to 5 mM in coupling buffer (0.2 M NaHCO₃, 0.5 M NaCl, pH 8.3 ± 0.1). Subsequently, the arginine solution was incubated with NHS magnetic beads overnight at 4 °C under gentle agitation. The pH was determined as 8.3 to promote deprotonation of the α‑amino group (pKa ~9.0) for efficient NHS-ester coupling, and to keep the side-chain guanidinium group (pKa ~12.5) protonated and non-reactive. After coupling, the remaining active sites were blocked with ethanolamine. The beads were resuspended in 100 μL storage buffer and stored at 4 °C. Coupling efficiency was estimated by measuring the depletion of arginine from the supernatant. The calculated binding capacity was ~176 nmol of arginine per milligram of beads.

Total protein lysates of A2780 cells treated with high-concentration L-arginine for 48 h was collected, and the protein concentration was adjusted to 1 mg/mL using KCl-based binding/washing buffer (100 mM KCl, 20 mM HEPES-NaOH, pH 7.9, 10% glycerol, 0.1% NP-40, 1 mM MgCl₂, 0.2 mM CaCl₂, 0.2 mM EDTA, 1 mM DTT, and 0.2 mM PMSF). Subsequently, 200 μL of protein solution was added to 0.5 mg of arginine or leucine-coupled NHS magnetic (control) beads, and incubated for 4 h at 4 °C. The magnetic beads were washed twice with a binding/washing buffer and then mixed with a 1 or 5 mM arginine solution for 1 h to elute the binding protein. Both the eluate (supernatant containing competitively eluted proteins) and the beads (containing any remaining tightly bound proteins) were collected separately. The beads were then boiled in 1× SDS-PAGE loading buffer. Finally, all samples (input, flow-through, wash, eluate fractions) were analyzed by SDS-PAGE and silver staining. The LC-MS identification and analysis were performed by Shanghai Bioprofile Technology Co., Ltd. (Shanghai, China).

### Immunoprecipitation (IP)

For immunoprecipitation, Pierce™ Classic Magnetic IP/Co-IP Kit (Cat. No.88804, Thermo Fisher Scientific, Waltham, USA.) was used according to manufacturer instructions. First, cell lysates containing 500 µg of total protein were incubated with 2 µg of the CRM1 antibody or rabbit IgG in 500 µL of IP Lysis/Wash Buffer overnight at 4 °C with rotation. Next, pre-washed Protein A/G Magnetic Beads (0.25 mg) were added to the antigen-antibody complex and incubated at room temperature for 1 h. The beads were collected magnetically and washed three times with 500 µL of IP Lysis/Wash Buffer, followed by washing with 500 µL of pure water. Binding proteins were eluted with 100 µL of 1× SDS-PAGE Sample Buffer (heated at 100 °C for 10 min) for downstream western blot analysis.

### Immunofluorescence

The A2780 cells were seeded into confocal dishes and treated as indicated. After 48 h in culture, the cells were washed twice with PBS to remove residual medium. Cells were fixed with 4% paraformaldehyde for 30 min and permeabilized with 0.5% Triton X-100 for 10 min. The cells were blocked with 5% BSA for 30 min and then incubated with FLAG or DDX3X primary antibodies at 4 °C overnight. For the DNA damage assay, cells were stained with the γH2AX primary antibody at 4 °C overnight. Then cells were incubated with a fluorescent secondary antibody at room temperature for 1 h. The nuclei were stained with DAPI for 15 min, mounted with antifade mounting medium (Cat. No. AP0271S, AccuRef Scientific, Xi’an, China), and imaged using an Olympus FV3000 laser scanning confocal microscope (Tokyo, Japan). Fluorescence images were analyzed using ImageJ software. Briefly, the DAPI channel was thresholded to create a nuclear mask. A cytoplasmic mask for each cell was generated by subtracting the nuclear mask area from the whole-cell area, which was defined by thresholding the target protein channel. The fluorescence intensity of the target protein within the nuclear and cytoplasmic masks was measured after background subtraction. The nuclear-to-cytoplasmic ratio (NCR) was then calculated for each individual cell.

### Scanning electron microscope

A2780 cells were fixed with 3% glutaraldehyde after adhering to coverslips. The specimens were washed with distilled water, followed by re-fixation with 1% osmium tetroxide. The samples were dehydrated using a graded ethanol series, critical point dried, and sputter-coated with gold. Imaging was performed using a JEOL JSM-IT700HR scanning electron microscope.

### Comet assay

The comet assay kit (Cat. No. C2041M, Beyotime, Shanghai, China) was used to detect DNA damage in single cells. First, cells treated with 10 µM cisplatin (Cat. No. T1564, TargetMol, Shanghai, China) or 1 mM H₂O₂ were harvested, counted, and resuspended in PBS at a density of ~1 × 10⁵ cells/mL. Subsequently, a comet assay slide (Cat. No. BS-CES-03A, Biosharp, Beijing, China) were precoated with a thin layer of 1% normal melting point agarose and air-dried. Cells were then mixed with pre-warmed 0.7% low melting point agarose, and then the mixture was immediately pipetted onto the precoated slide. The slides were subjected to lysis in a solution containing 10% DMSO at 4 °C for 2 h. Next, the glass slides were immersed in alkaline electrophoresis buffer (1 mM EDTA, 200 mM NaOH, pH ~13) and incubated at room temperature for 1 h to allow DNA unwinding. Electrophoresis was performed in an ice bath at a constant voltage of 25 V for 25 min. Finally, a neutral buffer (0.4 M Tris-HCl, pH 7.5) was used for neutralization, and a propidium iodide solution was used for staining, followed by observation under a fluorescence microscope. DNA damage was quantified by analyzing comet assay images using the Comet Assay Software Project (CASP) 1.2.2 software. For each experimental condition, at least 50 randomly selected and non-overlapping comets per slide from a minimum of three independent biological replicates were analyzed in a blinded manner. The software was used to automatically threshold and measure the comet head and tail for each cell. The primary metric for DNA strand break evaluation was the percentage of DNA in the tail (% Tail DNA). Additional parameters, including tail length and olive tail moment, were also recorded.

### RNA-seq

Trizol was used to separate and purify RNA from tissues and cells. Nanodrop ND-1000 (Nanodrop, Wilmington, Germany) was used to quantify and purify total RNA and to evaluate RNA integrity. Samples with concentration >50 ng/μL, RNA Integrity Number (RIN) value >7.0, and total RNA >1 μg were selected for downstream experiments. mRNA was captured using oligo(dT) magnetic beads, then fragmented, reverse transcribed, and amplified. An RNA library with a fragment size of 300 ± 50 bp was formed. Illumina Novase QTM 6000 (LC Biotechnology Co., Ltd., Hangzhou, China) was used for double-ended sequencing according to standard operations, and the sequencing mode was PE150. Reads were aligned, and quantification was performed using STAR software. Differential gene expression analysis was performed between the two different groups using the DESeq2 algorithm.

### CUT&Tag

The CUT&Tag (Cleavage Under Targets and Tagmentation) assay was performed using the AccuNext CUT&Tag Library Prep Kit for Illumina (Cat No. AG12556, Accurate Biology, China) according to the manufacturer’s instructions, with minor adaptations. Briefly, 1 × 10^6^ A2780 cells (viability >90%) per sample under the indicated treatments were immobilized on Concanavalin A (ConA)-coated magnetic beads. Cells were subsequently permeabilized with ice-cold Digitonin solution (provided in the kit) to enable nuclear access. Permeabilized cells were then sequentially incubated with a primary rabbit anti-DDX3X antibody (Cat. No. 11115-1-AP, Proteintech, Wuhan, China) and a secondary Goat Anti-Rabbit IgG antibody (Cat. No. AS070, ABclonal, Wuhan, China). A protein A/G-Tn5 transposome complex was subsequently added to bind the antibody-targeted sites. Tagmentation was initiated by adding Mg²⁺, enabling the targeted Tn5 transposase to simultaneously fragment the genomic DNA and ligate pre-loaded sequencing adapters (AccuNext Adapter Primers for Illumina). The reaction was stopped, and DNA fragments were released by Proteinase K digestion, followed by purification using magnetic beads. A final sequencing library was generated by PCR amplification for 16 cycles using the AccuNext PCR Mix II and index primers. The library was purified, quantified, and its size distribution was assessed using an Agilent Bioanalyzer prior to high-throughput sequencing on an Illumina platform.

### Metabolite extractions and LC-MS/MS Analysis

Targeted metabolomics was conducted using SHANGHAI APPLIED PROTEIN TECHNOLOGY (Shanghai, China) to quantitatively measure 650 metabolites related to amino acids, organic acids, carbohydrates, and fatty acids. Primary tumors and omental metastases were quickly frozen in liquid nitrogen immediately after dissection. Approximately 80 mg of the tissue was cut on dry ice and homogenized, and 800 μL of methanol/acetonitrile (1:1, v/v) extraction solvent was added to the homogenate for metabolite extraction. The mixture was centrifuged for 15 min (14000 × *g*, 4 °C), and the supernatant was dried in a vacuum centrifuge. For LC-MS analysis, the samples were re-dissolved in 100 μL acetonitrile/water (1:1, v/v) solvent, and **t**he supernatant was injected for analysis.

### Arginine content detection

An arginine content detection kit (cat. No. BC5630, Solarbio, Beijing, China) was used to determine the arginine content in the primary and metastatic tissues. In short, 100 mg of tissue was homogenized with 1 mL of Extraction Buffer I and centrifuged at 4 °C, 12,000 × *g* for 10 min. Subsequently, 800 μL of the supernatant was mixed with 150 μL of Extraction Buffer II and centrifuged. Reaction reagents were sequentially added to the blank tube, standard tube (containing 62.5 μmol/mL arginine), and sample tube. The absorbance of the sample at 525 nm was determined by a Bio-Tek SynergyLX microplate reader (Bio-Tek, Winooski, USA).

### Detection of ROS

The relative levels of reactive oxygen species (ROS) in tissue samples were quantified using a ROS assay kit (Cat. No. G1746, Servicebio, Wuhan, China) based on the dihydroethidium (DHE) fluorescence method. Briefly, fresh primary tumor and omental metastases (50 mg) from patients were rinsed with PBS and thoroughly homogenized in 450 μL of ice-cold homogenization buffer. The homogenate was then centrifuged at 10,000 × *g* for 10 min at 4 °C to collect the supernatant. For the assay, 10 μL of the tissue supernatant was combined with 90 μL of homogenization buffer and 1 μL of 100× DHE staining working solution per well in a black 96-well plate. After gentle mixing, the plate was incubated at 37 °C in the dark for 20 min. Fluorescence intensity was measured using a microplate reader with excitation at 520 nm. The final ROS level for each sample was normalized to its total protein content.

### Molecular docking and dynamics simulation

Molecular docking of L-arginine (HMDB ID: HMDB0000517) to human DDX3X protein (PDB:5E7J) was performed using Discovery Studio software (Accelrys Inc., California, USA). The structure of DDX3X was obtained by removing the original ligands and water molecules, followed by adding hydrogen atoms and assigning charges to the appropriate sites. Receptor-ligand docking was performed using the CDOCKER protocol with the default parameters. The molecular docking-derived complex with the lowest binding energy was subsequently subjected to molecular dynamics (MD) simulation using GROMACS 2022 to assess its structural stability. The protein-ligand complex was parameterized with the CHARMM36 force field for the protein and the GAFF2 force field for L-arginine. The system was solvated in a cubic periodic box with CHARMM-modified TIP3P water molecules, maintaining a minimum distance of 1.0 nm between the solute and the box boundary. Counterions were added to neutralize the system. Long-range electrostatic interactions were calculated using the Particle Mesh Ewald (PME) method. Prior to production MD, the system underwent energy minimization and equilibration in three sequential steps: (1) energy minimization using the steepest descent algorithm for 100,000 steps; (2) a 1,000,000-step (2 fs/step) NVT equilibration at 298 K using the Berendsen thermostat; (3) a 1,000,000-step (2 fs/step) NPT equilibration at 1 bar using the Parrinello-Rahman barostat. Following equilibration, an unconstrained production MD simulation was conducted for 100 ns with a 2-fs time step at constant temperature (298 K, V-rescale thermostat) and pressure (1 bar, Parrinello-Rahman barostat). Trajectory coordinates were saved every 10 ps for subsequent analysis. The stability of the complex was evaluated by calculating the root mean square deviation (RMSD), root mean square fluctuation (RMSF), radius of gyration (Rg), and solvent-accessible surface area (SASA). The binding free energy landscape and per-residue energy decomposition were also computed using the molecular mechanics/generalized born surface area (MM/GBSA) method. All structural visualizations and analyses were performed using PyMOL (v.2.5) and VMD (v.1.9.3).

### Surface plasmon resonance (SPR) analysis

SPR analysis was performed on a Cytiva Biacore 1 K instrument to evaluate the binding affinity of DDX3X to arginine. Purified DDX3X protein was immobilized on a CM5 sensor chip via standard amine coupling in sodium acetate buffer (pH 4.0), resulting in a final immobilization level of approximately 7498.3 response units (RU). Serial dilutions of the analyte (arginine) were sequentially injected over the protein surface at a constant flow rate. Binding kinetics and affinity were analyzed in real-time, and the sensorgrams were processed using the Biacore Insight Evaluation software (version 5.0.18) to determine the respective dissociation constants.

### Statistics and analysis

All data were presented as the mean ± standard error of the mean (SEM) or standard deviation (SD), as indicated in the figure legends. Each experiment was independently repeated at least three times. For comparisons between two groups, statistical significance was assessed using the unpaired two-tailed Student’s *t*-test. Comparisons among more than two groups were analyzed by one- or two-way ANOVA, followed by Dunnett’s (one-way) or Bonferroni (two-way) post hoc tests. Survival analysis was evaluated using the log-rank test, with curves plotted using the Kaplan–Meier method. Associations between continuous variables were quantified using Pearson’s correlation. Sample sizes were indicated in the respective figure legends. A minimum of three independent biological replicates were used for in vitro studies. For in vivo experiments, group sizes were guided by the established variability in tumor growth for the model, ensuring the statistical power to detect significant differences while adhering to the principle of reducing animal use. No samples or animals were excluded from analysis. All analyses and graphs were performed using GraphPad Prism 9.0.0 (GraphPad Software Inc., California, USA) and R software (4.5.1).

## Results

### Abnormal arginine metabolism in omental metastases of EOC

**Fig. 1:**
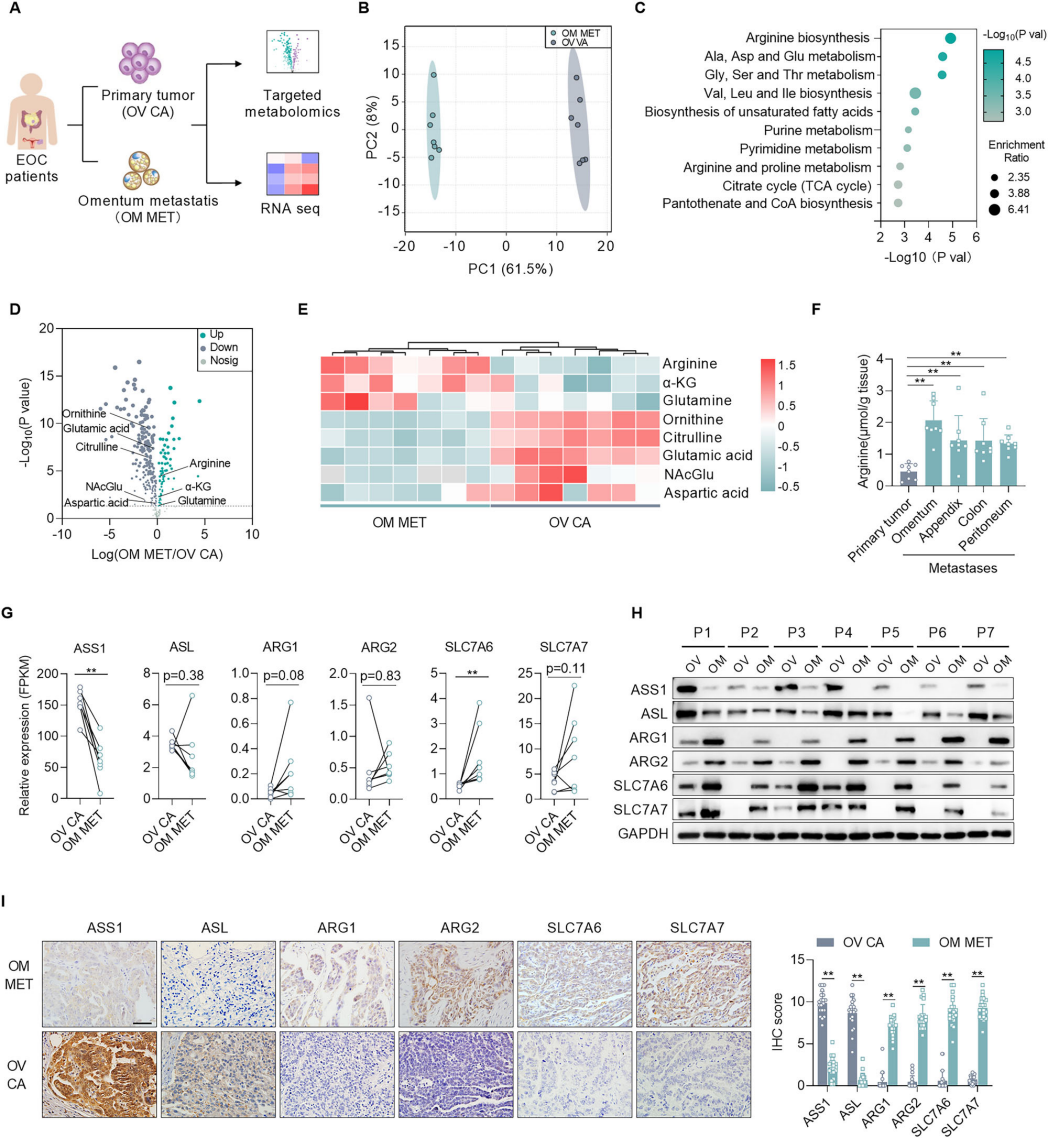
Abnormal arginine metabolism in omental metastases of EOC. **A** Schematic diagram of sample collection and analytical workflow. **B** Principal component analysis of metabolites detected in paired primary tumors and omentum metastasis (*n* = 7/group) by targeted metabolomics. **C** KEGG pathway analysis of the differentially metabolites between primary tumors and omentum metastasis. **D**, **E** A volcano plot and hierarchical clustering heatmap of arginine biosynthesis pathway-related metabolites in primary tumors and omentum metastasis (*n* = 7/group). **F** Quantification of arginine concentrations in primary tumors and metastatic lesions (omentum, appendix, colon, and peritoneum) using biochemical assay kit (*n* = 8). **G** mRNA expression of arginine metabolism-related enzymes (ASS1, ASL, ARG1, and ARG2) and transporter (SLC7A6 and SLC7A7) in EOC primary tumors and omentum metastases (*n* = 7) by RNA sequencing. **H** WB analyses for the protein level of ASS1, ASL, ARG1, ARG2, SLC7A6, SLC7A7, and GAPDH in primary tumors and omentum metastasis (*n* = 7). **I** Representative image and quantification of hematoxylin and eosin (H&E) and immunohistochemical staining for ASS1, ASL, ARG1, ARG2, SLC7A6, and SLC7A7 in primary tumors and omentum metastasis (*n* = 20). Scale bars, 50 μm. Data were shown as the mean ± SD (**F**, **G**) or mean ± SEM (**I**). The *p* value was calculated using one-way ANOVA (**F**), paired Student’s *t*-test (**G**), and unpaired two-tailed Student’s *t*-test (**I**). *p < 0.05, **p < 0.01.

To identify the metabolic characteristics of omental metastases, we analyzed primary ovarian tumors and omental metastases in seven patients with TP53 wild-type EOC using targeted metabolomics (Fig. 1A and Fig. S1A). Remarkably, omental metastases exhibited distinct metabolic profiles compared to primary tumors, characterized by the upregulation of 76 metabolites and the downregulation of 201 metabolites (Fig. 1B, D). Pathway enrichment analysis showed that several amino acid metabolic pathways involving arginine, alanine, and glycine were dysregulated (Fig. 1C). Notably, the metabolites associated with the arginine biosynthesis pathway exhibited significant remodeling. Specifically, arginine was significantly accumulated in omental metastases, followed by α-KG and glutamine, while ornithine, citrulline, and glutamic acid were accumulated in primary tumors (Fig. 1D, E). To further confirm the observed trend of increased arginine abundance in metastatic foci, we quantified arginine levels in primary tumors and metastases in different tissues and confirmed that arginine was significantly enriched in metastatic foci compared with primary tumors (Fig. 1F). Interestingly, RNA sequencing revealed a decreased expression of argininosuccinate synthetase (ASS1, an arginine biosynthesis enzyme) and a mildly increased expression of arginase 1 (ARG1, an arginine catabolic enzyme) in omental metastases compared to primary tumors. In addition, the expression of solute carrier family 7, member 6 (SLC7A6, an arginine transporter) was upregulated (Fig. 1G). These findings were further validated at the protein level. WB analysis of the seven paired samples confirmed the decrease in ASS1 and revealed a concurrent reduction in argininosuccinate lyase (ASL). Conversely, protein levels of ARG1, arginase 2 (ARG2), and the arginine transporters SLC7A6 and solute carrier family 7, member 7 (SLC7A7) were elevated in omental metastases (Fig. 1H). Moreover, the expression trends of ASS1, ASL, ARG1, ARG2, SLC7A6, and SLC7A7 were consistently corroborated by IHC in a larger, independent EOC cohort (Fig. 1I). These findings indicated that arginine accumulation in omental metastases primarily depends on exogenous uptake rather than endogenous synthesis.

To further confirm this deduction, we analyzed 223,363 high-quality single-cell sequencing data samples from 14 ovarian cancer patients according to public data [21]. Compared to primary tumor cells, metastatic cells exhibited the characteristic of impaired synthesis and enhanced uptake of arginine. Specifically, expressions of the synthesis enzymes ASS1 and ASL were downregulated, while levels of the transporters SLC7A6 and SLC7A7 were upregulated. In contrast to bulk sequencing data, metastatic cells showed decreased ARG1 and ARG2, a difference potentially due to the low prevalence and expression levels of positive cells within the sampled tissue (Fig. S1B–D). Additionally, higher expression of ASS1, SLC7A6, and SLC7A7 was associated with poorer patient outcomes according to TCGA (Fig. S1E–G). Collectively, these results indicated that a large amount of arginine was taken up by EOC cells within omental metastases. Therefore, it is essential to investigate the role of arginine in EOC progression.

### Arginine deprivation inhibits the growth and metastases of EOC in vivo

**Fig. 2:**
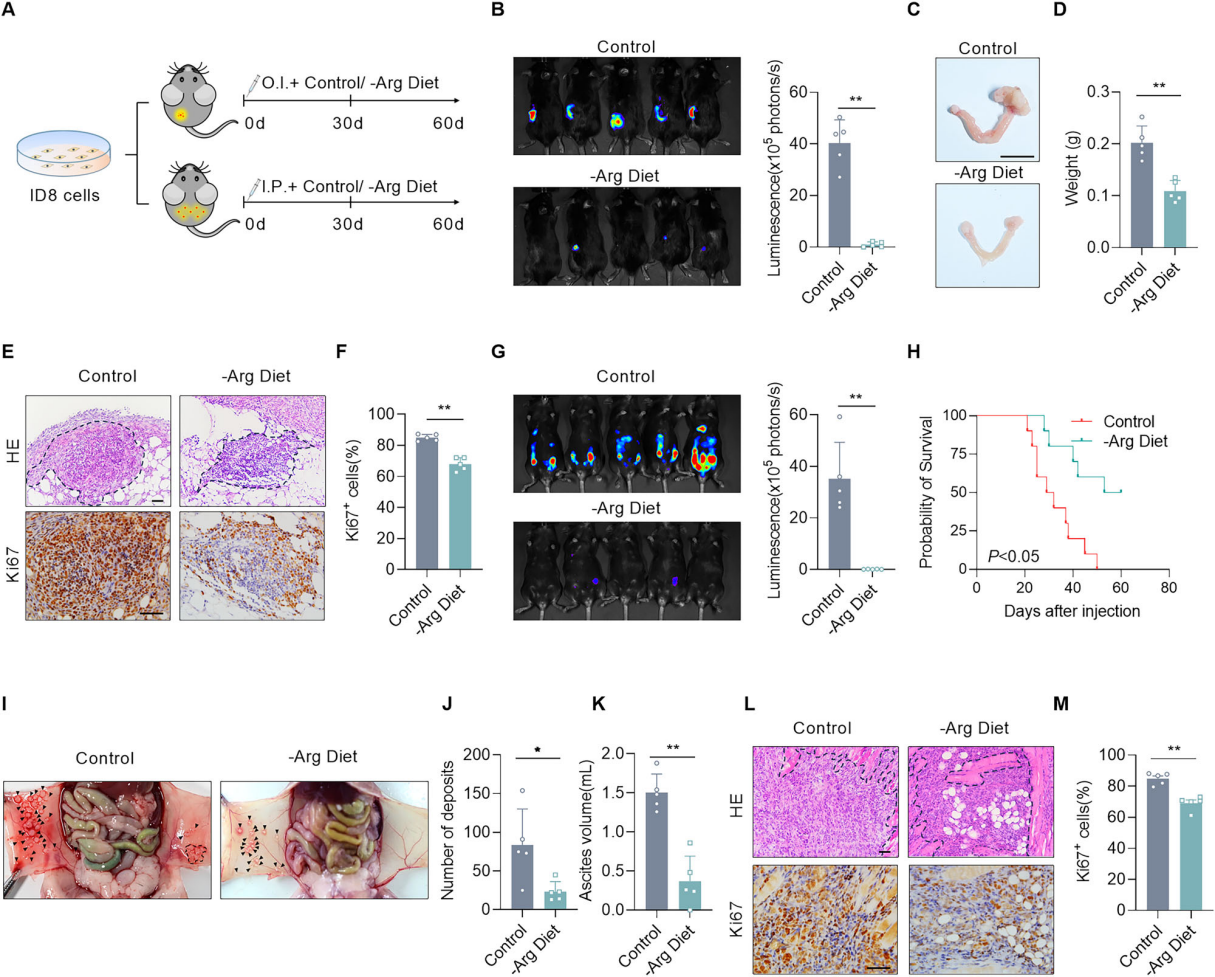
Arginine deprivation inhibits the growth and metastases of EOC in vivo. **A** Schematic illustrating the establishment of an orthotopic ovarian cancer model through intraovarian injection (OI) of ID8 cells (1 × 10^6^ cells/mouse) and intraperitoneal ovarian cancer model via intraperitoneal injection (IP) (1 × 10^6^ cells/mouse) in 8-week-old female C57BL/6 mice (*n* = 5/group). Mice were randomized to receive either standard chow or arginine restriction diet after implantation. **B** Representative IVIS bioluminescence images and quantitative analysis of bioluminescence signals of mice with indicated treatments at 30 days after orthotopic injection of ID8-Luc cells (*n* = 5/group). **C** Representative images of ovaries from orthotopic ovarian cancer model with indicated treatments at 60 days after injection (*n* = 5/group). Scale bars, 1 cm. **D** Weights of ovaries from orthotopic ovarian cancer model with indicated treatments (*n* = 5/group). **E**, **F** Representative images of H&E, Ki67 staining and percentage of Ki67-positive cells in tumors from orthotopic ovarian cancer model (*n* = 5/group). Scale bars, 50 μm. **G** Representative IVIS bioluminescence images and quantitative analysis of bioluminescence signals of mice with indicated treatments at 30 days after intraperitoneal injection of ID8-Luc cells (*n* = 5/group). **H** Kaplan–Meier analysis of mice intraperitoneally injected with ID8-Luc cells with the indicated diet (*n* = 10/group). **I**–**K** Representative images of peritoneal metastasis (**I**), total number of metastatic deposits (**J**), and ascites volume (**K**) from mice with the intraperitoneal ovarian cancer model with indicated treatments at 35 days after injection (*n* = 5/group). **L**, **M** Representative images of H&E, Ki67 staining and percentage of Ki67-positive cells of tumors from the intraperitoneal ovarian cancer model (*n* = 5/group). Scale bars, 50 μm. Data were shown as the mean ± SD. The *p* value was calculated using an unpaired two-tailed Student’s *t*-test (**B**, **D**, **F**, **G**, **J**, **K**, **M**). *p < 0.05, **p < 0.01.

To explore the effects of arginine on the occurrence and progression of EOC, we established an orthotopic EOC model based on a previous study [17]. First, we identified ID8 as a distinct cell line characterized by arginine with deficiency of endogenous synthesis as well as dependence on exogenous uptake (Fig. S2A, B). Meanwhile, analysis of ID8-derived primary tumors and metastases revealed that their expression profile closely resembled that observed in clinical patient samples (Fig. S2C and Fig. 1H). Subsequently, the mice were unilaterally and intraovarially injected with mouse EOC cell line ID8 expressing luciferase, and then fed an arginine-deprived diet or a common amino acid diet (Fig. 2A). 30 days after injection, bioluminescence imaging revealed that the orthotopic tumors of mice with arginine deprivation were significantly smaller than those of the control group (Fig. 2B). The tumor weights were also confirmed to be significantly lower in the arginine-deprived mice compared to the control group after 60 days, and the proportions of Ki67-positive cells corroborates the findings as well (Fig. 2C–F). Similarly, we established a disseminated EOC mouse model via intraperitoneal injection of ID8 cells (Fig. 2A) [22]. 30 days after injection, the bioluminescence of cells in the abdominal cavity was significantly lower in arginine-deprived mice than in the control group (Fig. 2G). All of the mice fed with the control diet died within two months. Conversely, the survival rate of the arginine-deprived mice was significantly better (Fig. 2H). Moreover, quite consistent results were obtained compared with the in-situ tumor model through dissection and Ki67 staining (Fig. 2I–M). These results indicate that dietary arginine deprivation inhibits the progression and intraperitoneal metastasis of EOC.

### Supplementing or enhancing arginine uptake promotes the proliferation, migration, and invasion of EOC cells

**Fig. 3:**
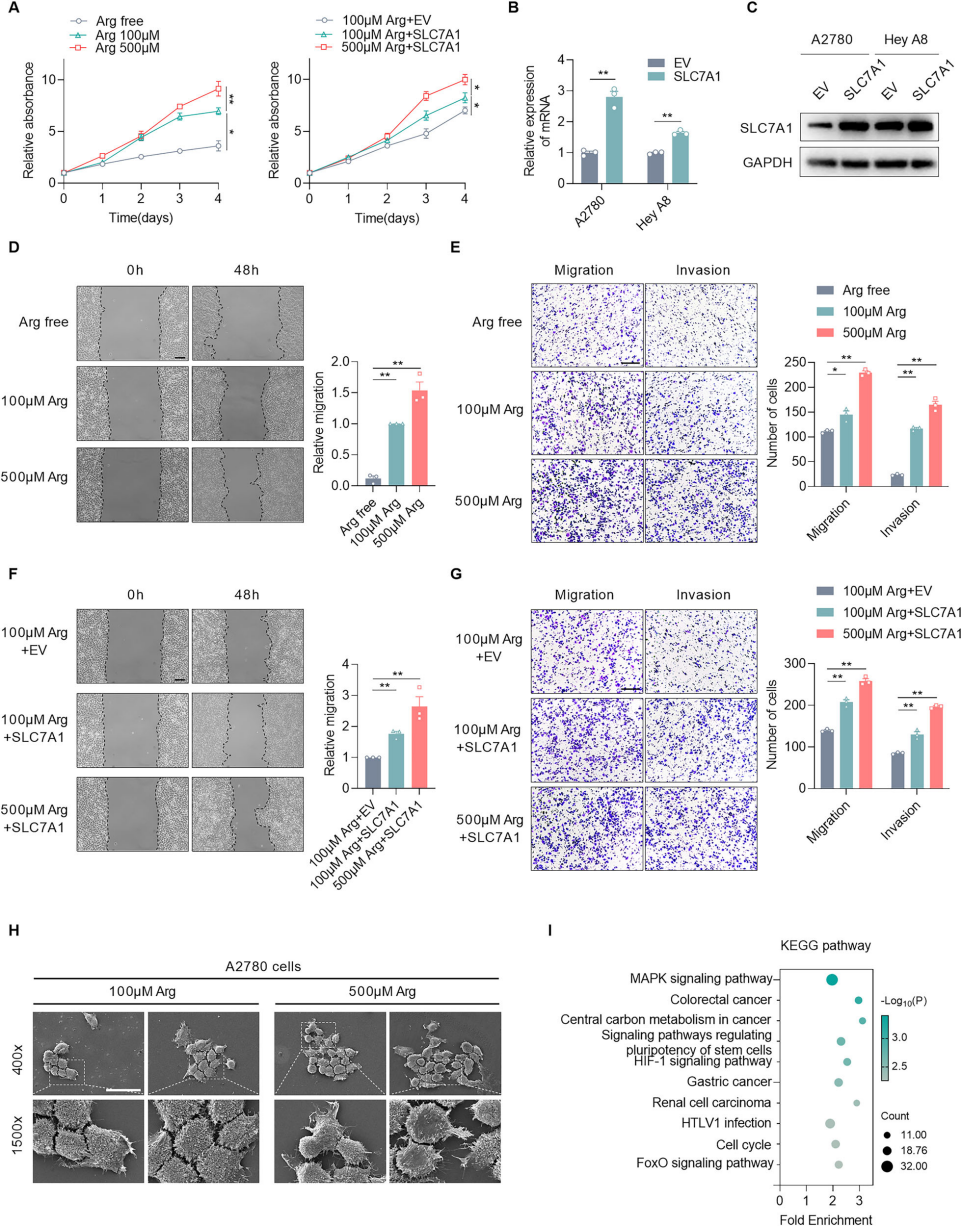
Supplementing or enhancing arginine uptake promotes the proliferation, migration, and invasion of EOC cells. **A** Cell proliferation was assessed by CCK-8 assay in A2780 cells with the indicated treatments. **B** Quantitative real-time polymerase chain reaction (RT-qPCR) analyses of SLC7A1 and GAPDH mRNA levels in A2780 cells and Hey A8 cells overexpressing SLC7A1. **C** WB analyses of SLC7A1 and GAPDH protein levels in A2780 cells and Hey A8 cells overexpressing SLC7A1. **D** Representative images of wound-healing assay on A2780 cells under the indicated treatments. Scale bars, 100 μm. **E** Transwell assays showing migration and invasion abilities for A2780 cells with treatments as indicated. Scale bars, 100 μm. **F** Representative images of wound-healing assay on A2780 cells with treatments as indicated. Scale bars, 100 μm. **G** Transwell assays indicating migration and invasion abilities for A2780 cells with treatments as indicated. Scale bars, 100 μm. **H** Scanning electron microscope images of A2780 cells treated with 100 μM and 500 μM arginine. Scale bars, 5 μm. **I** KEGG enrichment analysis of differently expressed genes in A2780 cells treated with 100 and 500 μM arginine. Data were shown as the mean ± SEM. The *p* value was calculated using two-way ANOVA (**A**), one-way ANOVA (**D**–**G**), and unpaired two-tailed Student’s *t*-test (**B**). *p < 0.05, **p < 0.01.

To verify the crucial role of exogenous arginine in the malignant development of EOC, we investigated the effects of arginine supplementation in vitro. First, we evaluated the levels of arginine-related metabolic enzymes in several human EOC cell lines using the Human Protein Atlas (HPA) database. Considering that A2780 and HEY A8 cells, which possess characteristics similar to those of omental metastases in EOC, appeared to be defective in the endogenous synthesis of arginine, we chose these two cell lines for subsequent experiments (Fig. S3A). To better simulate physiological conditions, we first measured arginine concentrations in peripheral blood samples from five EOC patients with omental metastases and five age-matched healthy individuals. The results showed that circulating arginine levels in healthy individuals ranged from 53 to 118 μM, whereas the concentrations in EOC patients with metastases were markedly higher, ranging from 144 to 487 μM (Fig. S3B). Meanwhile, the arginine levels detected in healthy controls were consistent with the physiological ranges (e.g., Human Metabolome Database (HMDB), 60–100 μM in adult blood), yet remained quite below the concentration present in standard RPMI 1640 culture medium (PROCELL, 1.15 mM). Therefore, 100 μM was regarded as the physiological concentration of arginine, while 500 μM was regarded as a high concentration. The CCK-8 assay revealed that the cell proliferation rate was slower in the arginine-deprived medium compared to the medium with a higher concentration of arginine (Fig. 3A). Besides, the capacity of cell invasion and migration becomes stronger as the concentration of arginine becomes higher (Fig. 3D, E). Additionally, the expression level of SLC7A1 was found to be higher in EOC compared to the normal tissue across multiple datasets (Fig. S3C). To further confirm the effect of SLC7A1, SLC7A1-overexpressed cells were constructed and verified, with mRNA levels increased by 2.8-fold in A2780 cells and 1.6-fold in Hey A8 cells relative to their controls, respectively (Fig. 3B, C). Then we found that the capacities of cell proliferation, migration, and invasion were enhanced when SLC7A1 was overexpressed (Fig. 3A, F, G). The variation of the aggressive phenotype was also verified in HEY A8 cells (Fig. S3D–H). Meanwhile, SLC7A1 knockdown significantly suppressed the aggressive phenotypes (Fig. S4A–F). Furthermore, scanning electron microscopy showed that the number of pseudopodia in A2780 cells increased when the arginine concentration was elevated (Fig. 3H). Additionally, RNA-seq data demonstrated that the MAPK signaling pathway was significantly activated in A2780 cells, accompanied by high concentrations of arginine, and the upregulated genes included VEGFA, MYC, TGFB2, and FOS, which indicated that arginine plays a vital role in promoting the aggressive progression of EOC (Fig. 3I and Fig. S3I). These findings imply that supplementing or enhancing arginine uptake promotes the proliferation, migration, and invasion of EOC cells.

### Arginine binds to RNA helicase DDX3X in EOC cells

**Fig. 4:**
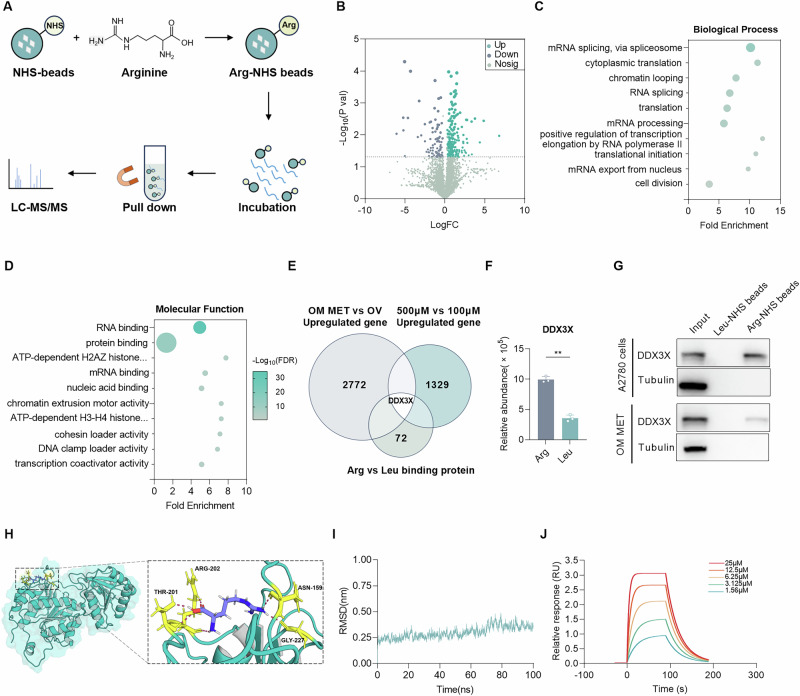
Arginine binds to RNA helicase DDX3X in EOC cells. **A** Scheme of L-arginine-immobilized NHS magnetic beads and the enrichment strategy for arginine-binding proteins. **B** Volcano plot of arginine- and leucine-binding proteins. **C**, **D** GO analysis of arginine-binding proteins in A2780 cells. **E** Venn diagram showing arginine-binding proteins that were upregulated in both 500 μM arginine-treated A2780 ovarian cancer cells and omentum metastases. **F** Relative abundance of DDX3X protein captured by arginine or leucine-coupled magnetic beads. **G** WB detection of DDX3X in A2780 cells with arginine treatment, or lysate from omentum metastasis, and elution after purification with L-leucine- or L-arginine-immobilized NHS magnetic beads. **H** Molecular docking between DDX3X (HMDB ID: HMDB0000517) and L-arginine (PubChem CID: 6322) performed using Discovery Studio. **I** Molecular dynamics (MD) simulation was performed using Gromacs. The backbone root mean square deviation (RMSD) of the DDX3X-L-arginine complex over the 100 ns MD simulation was shown. **J** Surface plasmon resonance analysis for the binding of L-arginine to DDX3X. Data were shown as the mean ± SD. The *p* value was calculated using an unpaired two-tailed Student’s *t*-test (**F**). **p < 0.01.

Arginine often interacts with specific proteins to transcriptionally regulate the expression of related genes. For example, various transcription factors can influence the metabolic adaptability and survival capacity of T cells by sensing arginine levels [23]. In hepatocellular carcinoma cells, RBM39 interacts with arginine to transcriptionally control the expression of genes regarding tumorigenesis and metabolism [14]. Consequently, we hypothesized that arginine promotes tumor progression by interacting with its binding protein in EOC. To identify the potential binding protein, a pull-down experiment was performed using arginine-coupled NHS magnetic beads (Fig. 4A and Fig. S5A). Then, 4013 proteins were identified through mass spectrometry (Fig. 4B and Fig. S5B). Compared with previous studies, we found that the enriched proteins included most of the known arginine-binding proteins (Fig. S5C) [14, 23]. Such proteins as RBM39, DEAD-box helicase 5 (DDX5), DEAD-box helicase 17 (DDX17), and receptor for activated C kinase 1 (RACK1) were validated to bind with arginine (Fig. S5D, E). Enrichment analyses indicated that arginine-binding proteins were predominantly involved in biological processes, such as RNA splicing, RNA processing, and cytoplasmic translation, as well as molecular functions, including RNA and protein binding (Fig. 4C, D). Based on these findings, we focused on RNA-binding proteins and selected the top ten differentially expressed candidates for further analysis (Fig. S5F). Combined with RNA sequencing, we found that only DDX3X was highly expressed in omental metastases or A2780 cells treated with a high concentration of arginine, which has been found overexpressed in primary EOC in the GEO database, yet little is known about its role in metastasis (Fig. 4E, F). Subsequently, we verified that arginine may bind to DDX3X in omental metastatic tissue or A2780 cells with a high concentration of arginine (Fig. 4G). Thus, molecular docking was implemented to find that arginine binds to the catalytic center of DDX3X via hydrogen bonds. Specifically, the interactions between arginine and DDX3X seem most likely at positions N159, T201, R202, and G227 (Fig. 4H). Using the highest-scoring docking pose as the initial structure, we performed molecular dynamics simulations to evaluate the stability of the complex. The results showed that the root mean square deviation (RMSD) fluctuated within a range of 0.2–0.5 nm during the simulation, and the overall binding free energy was −14.62 kcal/mol (Fig. 4I). These data collectively demonstrate that the DDX3X-L-arginine interaction is dynamically stable. Furthermore, surface plasmon resonance (SPR) analysis revealed that arginine indeed binds to DDX3X with a steady state affinity KD of 2.15 µM (Fig. 4J).

DDX3X contains a helicase core that is common to all DEAD-box proteins and is flanked by two intrinsically disordered regions (IDRs) [24, 25]. To investigate the binding site of DDX3X that arginine interacts to, we constructed a variety of DDX3X truncations, including the deletion of N-terminal intrinsically disordered regions (△N-IDR), the helical core domain (△Helicase), and the C-terminal intrinsically disordered regions (△C-IDR) (Fig. S5G). Using arginine-coupled NHS magnetic beads, we enriched proteins from cells transfected with these truncations and found that deleting the N-IDR of DDX3X abolished arginine binding (Fig. S5H). The results indicated that arginine interacts with DDX3X within its N-IDR. Moreover, site-directed mutagenesis demonstrated that arginine binds specifically to DDX3X at Asn159 in EOC cells (Fig. S5I).

### Arginine promotes DDX3X nuclear retention by inhibiting NES in EOC

**Fig. 5:**
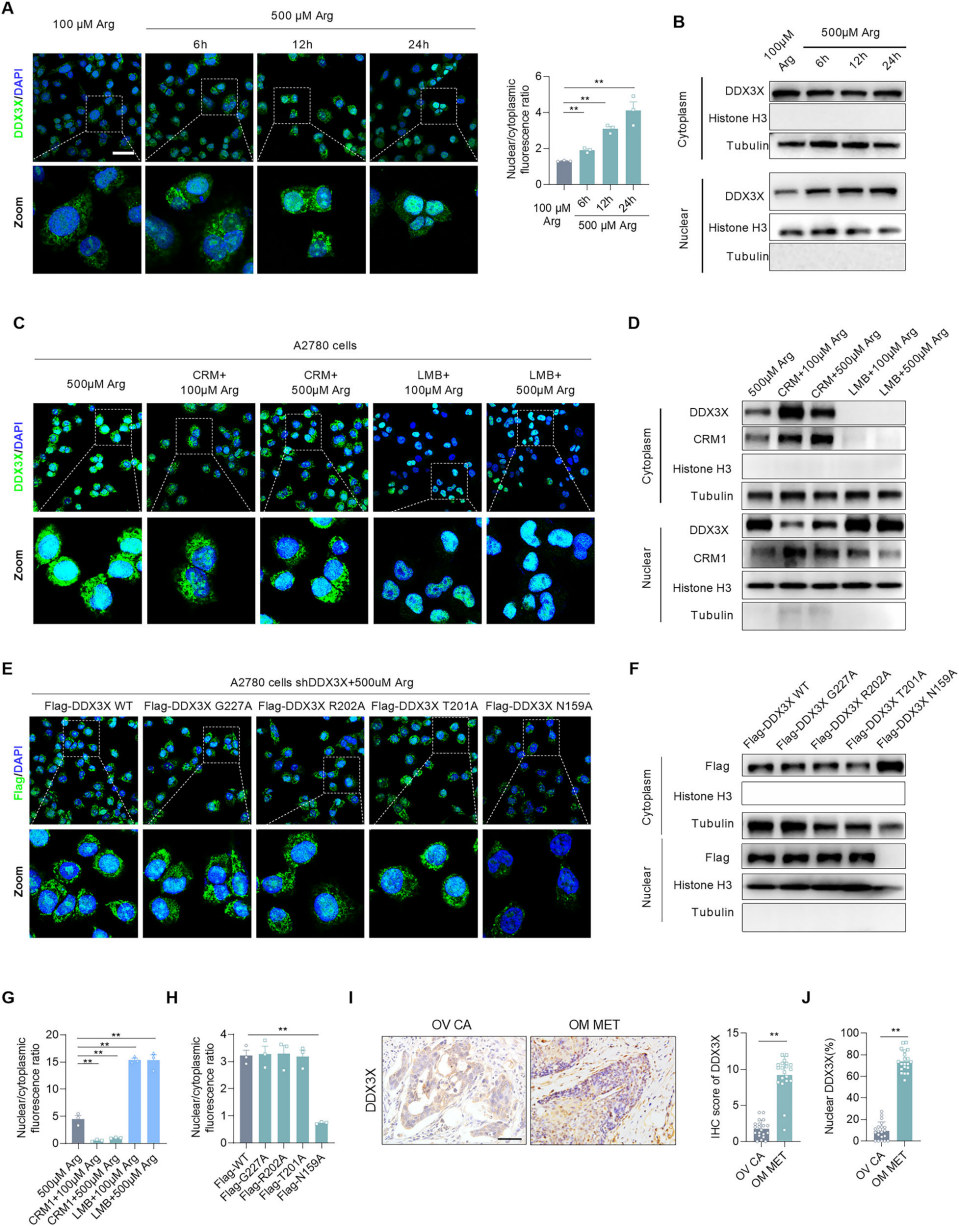
Arginine promotes DDX3X nuclear retention by inhibiting NES in EOC. **A** Representative confocal microscopy images and immunofluorescence analysis of DDX3X in A2780 cells treated with arginine at the indicated times. Scale bars, 10 μm. **B** WB analyses of DDX3X, histone H3, and tubulin protein levels in cytoplasmic and nuclear fractions of A2780 cells under the indicated treatments. **C** Representative confocal microscopy images of DDX3X immunofluorescence in A2780 cells treated as indicated. Scale bars, 10 μm. **D** WB analyses of DDX3X, CRM1, histone H3, and tubulin protein levels in the cytoplasmic and nuclear fractions of A2780 cells under the indicated treatments. **E** Representative confocal microscopy images of Flag immunofluorescence in A2780 shDDX3X cells treated as indicated. Scale bars, 10 μm. **F** WB analyses of Flag, histone H3, and tubulin protein levels in the cytoplasmic and nuclear fractions of A2780 shDDX3X cells under the indicated treatments. **G** Quantification of the nuclear-to-cytoplasmic ratio of DDX3X fluorescence intensity in A2780 cells treated with arginine as indicated. **H** Quantification of the nuclear-to-cytoplasmic ratio of DDX3X fluorescence intensity in A2780 shDDX3X cells treated with arginine as indicated. **I** Representative images and quantification of immunohistochemical staining of DDX3X in primary tumors and omentum metastases (*n* = 20). Scale bars, 50 μm. **J** Immunohistochemical quantification of nuclear DDX3X in primary tumors and omentum metastases (*n* = 20). Scale bars,50 μm. Data were shown as the mean ± SEM. The *p* value was calculated using one-way ANOVA (**A**, **G**, **H**), and unpaired two-tailed Student’s *t*-test (**I**, **J**). *p < 0.05, **p < 0.01.

Arginine plays crucial roles in various metabolic and physiological processes. It has been summarized that cytoplasmic proteins sense the concentration of arginine to regulate diverse cellular signaling pathways and functions [26]. To investigate the biological effects of arginine on DDX3X, immunofluorescence was used to observe the abundance and subcellular localization of DDX3X in A2780 cells. The results showed that nuclear accumulation of DDX3X increased significantly with rising concentration of arginine, whereas its cytoplasmic level remained largely unchanged (Fig. 5A). Immunoblotting also confirmed that arginine treatment enhanced the nuclear accumulation of DDX3X (Fig. 5B). Accordingly, we propose that arginine promotes DDX3X nuclear accumulation by binding to it.

Given that residues 1–22 of the DDX3X N-terminus contain a predicted nuclear export signal (NES) [27], we hypothesized that NES is associated with DDX3X nuclear accumulation mediated by arginine. Subsequently, immunofluorescence and WB analyses showed that overexpression of chromosome region maintenance 1/exportin 1 (CRM1) promoted the cytoplasmic accumulation of DDX3X and reduced its nuclear level, yet arginine treatment weakened the effect (Fig. 5C, D, G). However, treatment with leptomycin B (LMB), a specific CRM1 inhibitor, resulted in nuclear retention of DDX3X, while arginine treatment had no significant effect (Fig. 5C, D, G). Furthermore, co-immunoprecipitation (Co-IP) assays demonstrated that high concentrations of arginine inhibited the binding of CRM1 to DDX3X (Fig. S6A). These results collectively indicate that the binding of arginine to DDX3X inhibits the NES.

To identify the specific DDX3X residue responsible for arginine-enhanced nuclear retention, four DDX3X point mutants (N159A, T201A, R202A, and G227A) were individually transfected into shDDX3X cells, which were then treated with a high concentration of arginine. The results illustrated that the N159A mutation prevented arginine from inducing DDX3X nuclear retention (Fig. 5E, F, H). Meanwhile, a series of truncations targeting DDX3X N-IDR were constructed (Fig. S6B). We found that disrupting either the entire N-IDR or the predicted NES led to pronounced nuclear accumulation of DDX3X, whereas deleting residues 23–168 or the N159A mutation facilitated nuclear export of DDX3X (Fig. S6B–E). The findings indicate that arginine binds to the Asn159 site of DDX3X and promotes its nuclear retention by inhibiting NES.

By assessing DDX3X expression in clinical samples, we observed that its expression was predominantly nuclear and significantly higher in metastatic lesions compared to primary foci of EOC (Fig. 5I, J). Furthermore, survival analysis revealed that high DDX3X expression correlated with poor prognosis of EOC patients (Fig. S6F).

### Arginine facilitates EOC metastasis through DDX3X-mediated transcriptional enhancement of the DDR pathway

To explore the role of DDX3X in EOC progression, the expression of DDX3X was knocked down in A2780 cells, and the interference efficiency was confirmed (Fig. S7A). As anticipated, DDX3X knockdown inhibited the proliferation, migration, and invasion of EOC cells, as well as the pro‑tumor effects induced by a high concentration of arginine (Fig. S7B–F). We then expressed wild-type DDX3X or the N159A mutant in A2780 shDDX3X cells and treated the cells with a high concentration of arginine. Compared to wild-type DDX3X, expression of the N159A mutant impaired the proliferative, invasive, and migratory capacities of EOC cells. This finding demonstrates that arginine promotes EOC metastasis by binding to DDX3X at Asn159. (Fig. S7G–I).

**Fig. 6:**
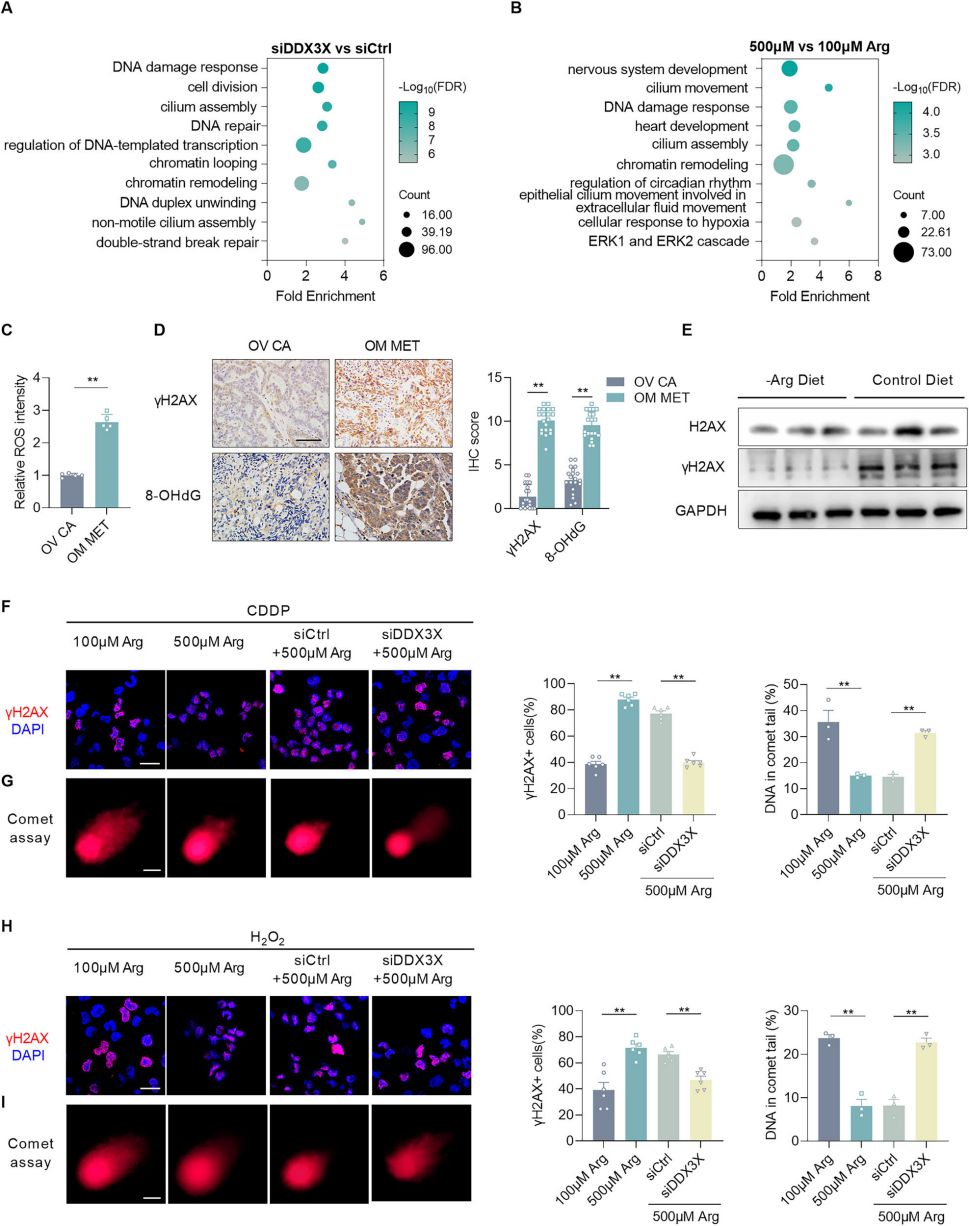
Arginine binds to DDX3X and facilitates DNA damage response. **A** GO analysis of differentially expressed genes between DDX3X knockdown and control A2780 cells. **B** GO analysis of differentially expressed genes between A2780 cells with 100 and 500 μM arginine. **C** The relative levels of reactive oxygen species (ROS) were measured using the dihydroethidium (DHE) fluorescence method in fresh primary tumor and omental tissues (*n* = 5). **D** Representative image and quantification of immunohistochemical staining of γH2AX and 8-OHdG in primary tumors and omentum metastases (*n* = 20). Scale bars, 50 μm. **E** WB analyses of the protein levels of H2AX, γH2AX, and GAPDH in metastases from mice fed with the indicated diets. **F** Representative images and quantification of γH2AX immunofluorescence staining in A2780 cells treated with cisplatin and under the indicated treatments. Scale bars, 10 μm. **G** Representative images and quantification of the comet assay in A2780 cells treated with cisplatin and under the indicated treatments. Scale bars, 10 μm. **H** Representative images and quantification of immunofluorescence staining of γH2AX in A2780 cells treated with H_2_O_2_ (1 mM final concentration) and under the indicated treatments. Scale bars, 10 μm. **I** Representative images and quantification of comet assay in A2780 cells treated with H_2_O_2_ (1 mM final concentration) and under the indicated treatments. Scale bars, 10 μm. Data were shown as the mean ± SEM. The *p* value was calculated using one-way ANOVA (**F**- **I**), and unpaired two-tailed Student’s *t*-test (**C**, **D**). *p < 0.05, **p < 0.01.

To uncover the underlying mechanism, we performed transcriptome sequencing (RNA-seq) on A2780 cells under different conditions. Notably, not only did DDX3X knockdown significantly affect biological processes, including DDR, but arginine treatment similarly upregulated the specific pathway (Fig. 6A, B). Consequently, we hypothesized that arginine may influence DDR through binding to DDX3X, thereby promoting the malignant progression of EOC. To further investigate the DNA damage status in omental metastases, we evaluated the extent and consequences of oxidative stress, which is the most common cause of DNA damage [28, 29]. The results showed a significant increase in the ROS level, as well as the expression of both gamma-H2A histone family member X (γH2AX, a marker of double-strand breaks) and 8-hydroxy-2’-deoxyguanosine (8-OHdG, a marker of oxidative DNA damage) in omental metastases versus primary tumors, revealing substantial and multifaceted DNA oxidative damage in metastatic foci (Fig. 6C, D). On the contrary, arginine deprivation reduced the expression of DDR-related γH2AX in the intraperitoneal EOC model (Fig. 6E). Beyond intrinsic factors like oxidative stress, cancer cells in clinical settings must overcome extrinsic pressures imposed by therapeutic interventions [30]. To verify whether arginine modulates the DDR process, we induced DNA damage in cells using cisplatin or hydrogen peroxide. As we expected, arginine supplementation significantly increased the proportion of γH2AX+ cells and shortened the length of the comet tail. Additionally, knockdown of DDX3X abolished the above effects (Fig. 6F–I). These findings imply that arginine facilitates the DDR process in metastatic EOC cells via DDX3X, which may represent an adaptive survival strategy, enabling EOC cells to confront microenvironmental stress.

Multiple pathways modulate the process of DDR, including non-homologous end joining, homologous recombination, mismatch repair, and nucleotide excision repair [30]. Subsequently, we investigated the differentially expressed molecules within the DDR pathway in cells treated with high-concentration arginine or DDX3X knockdown. The heatmaps depicted that DDR-related genes were upregulated following arginine treatment, but were downregulated after DDX3X knockdown (Fig. S8A). Given the role of nucleus-retained DDX3X in promoting the DDR pathway, we speculate that DDX3X may function to transcriptionally regulate DDR-related genes. To directly examine whether DDX3X binds to the promoter regions of these genes, we performed CUT&Tag profiling under high- versus low-arginine conditions. The results revealed increased occupancy of DDX3X at the promoter regions following high-arginine treatment (Fig. S8B, C). Enrichment analysis of genes associated with differential peaks highlighted key biological processes, including RNA polymerase II-mediated transcription, chromatin remodeling, cellular senescence, and DDR (Fig. S8D). Importantly, a high concentration of arginine facilitated DDX3X binding to the promoters of several DDR genes, including ataxia-telangiectasia mutated (ATM), ataxia-telangiectasia and Rad3-related (ATR), checkpoint kinase 2 (CHK2), Poly (ADP-ribose) polymerase 1 (PARP1), RAD18 E3 ubiquitin protein ligase (RAD18), and MutS homolog 2 (MSH2) (Fig. S8E). These findings offer additional support for DDX3X as a direct transcriptional regulator of DDR-related genes.

**Fig. 7:**
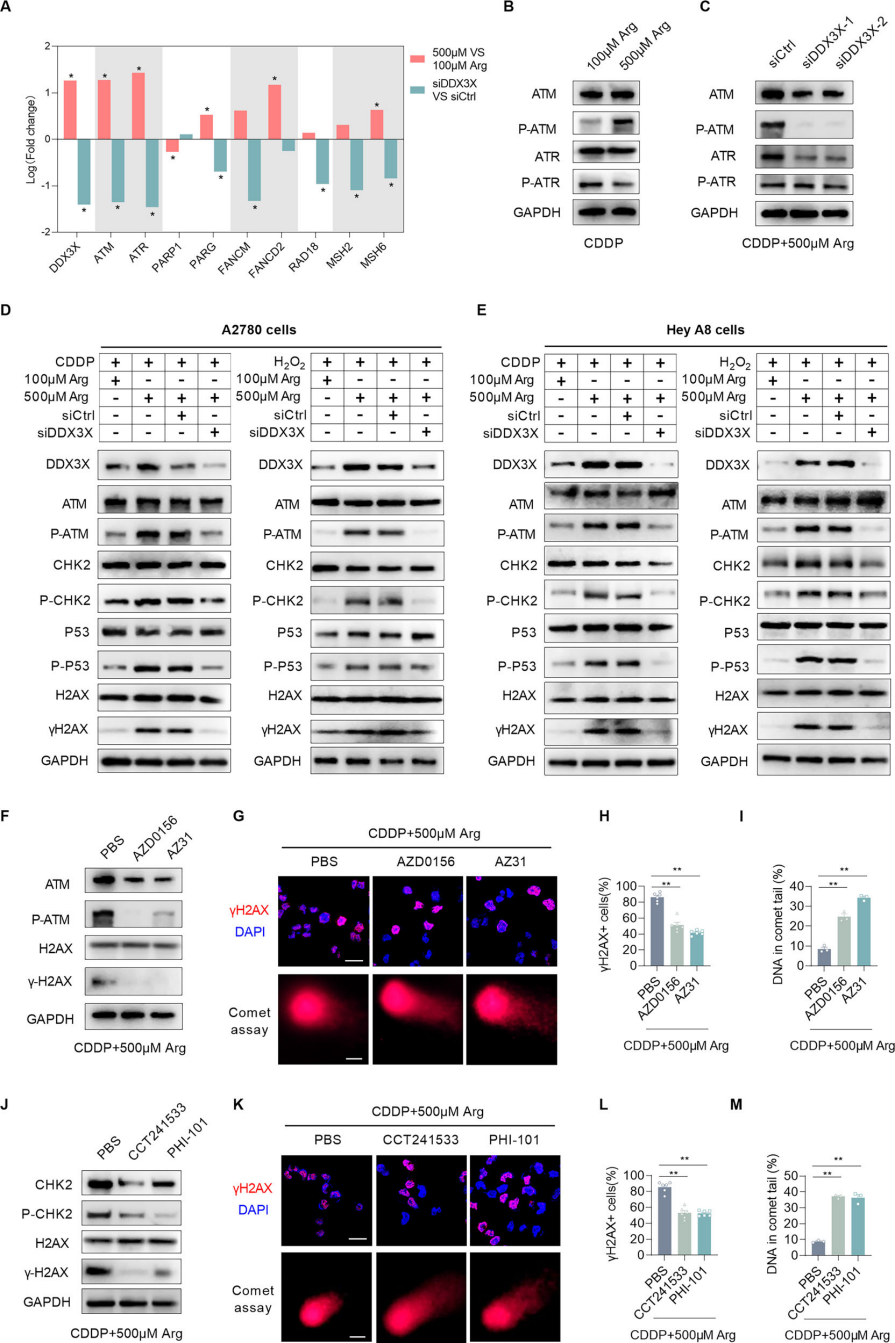
Arginine facilitates the DNA damage response by activating the ATM/CHK2/p53 axis. **A** The bar graph shows the fold changes in DDR gene expression following treatment with 500 μM arginine or DDX3X knockdown. **B**, **C** WB analyses for the protein level of ATM, Phospho-ATM (Ser 1981), ATR, Phospho-ATR, and GAPDH in metastasis of mice treated with the indicated diets. **D**, **E** WB showing the protein level of DDX3X, ATM, Phospho-ATM (Ser 1981), CHK2, Phospho-CHK2 (Thr68), P53, Phospho-P53 (Ser15), H2AX, γH2AX, and GAPDH in A2780 or HEY A8 cells with indicated treatments. **F** WB analyses for the protein level of ATM, Phospho-ATM (Ser 1981), H2AX, γH2AX, and GAPDH in A2780 cells with indicated treatments. **G**–**I** Representative images and quantification of immunofluorescence staining of γH2AX, comet assay in A2780 cells treated with cisplatin and ATM inhibitors (AZD0156[10 μM] and AZ31[5 μM]). Scale bars, 10 μm. **J** WB analyses for the protein level of CHK2, Phospho-CHK2 (Thr68), H2AX, γH2AX, and GAPDH in A2780 cells with the indicated treatments. **K**–**M** Representative images and quantification of immunofluorescence staining of γH2AX, comet assay in A2780 cells treated with cisplatin and CHK2 inhibitors (CCT241533 [5 μM] and PHI-101[1 μM]). Scale bars, 10 μm. Data were shown as the mean ± SEM from three independent experiments. The *p* value was calculated using one-way ANOVA (**H**, **I**, **L**, **M**). *p < 0.05, **p < 0.01.

According to the transcriptome analysis, ATM and ATR emerged as the most significantly altered among DDR-related genes in response to either arginine treatment or DDX3X knockdown, thus positioning the ATM/ATR pathway as a key node through which DDX3X regulate DDR (Fig. 7A). In CDDP-treated cells, we confirmed that arginine treatment robustly enhanced the ATM protein level of both total and phosphorylated (Ser 1981) form, whereas ATR was barely affected (Fig. 7B). And DDX3X knockdown further suppressed ATM phosphorylation (Fig. 7C). These findings indicated that DDX3X primarily regulate DDR via the ATM pathway. Given that ATM-CHK2 signaling sustains p53 phosphorylation during cellular stress and DNA damage, we assessed the correlation of DDX3X level with the expression level of ATM, CHK2, TP53, and H2AX in the TCGA cohort. Correlation analysis revealed significant positive associations between the expression levels of DDX3X and ATM, CHK2, and TP53 (Fig. S9A–D). Furthermore, WB analysis confirmed that arginine supplementation promoted the phosphorylation of ATM, CHK2, and TP53, whereas DDX3X knockdown suppressed their activation and thereby impeded DDR (Fig. 7D, E). To functionally validate that arginine promotes DDR through the ATM/CHK2/P53 axis, we employed two distinct ATM inhibitors (AZD0156 and AZ31) in CDDP- and arginine-treated A2780 cells. The results showed that ATM inhibition blocked the arginine-conferred cyto-protection against CDDP-induced cytotoxicity (Fig. 7F–I). Similarly, CHK2 inhibitors (CCT241533 and PHI-101) gave rise to a consistent effect (Fig. 7J–M). Meanwhile, IHC of clinical samples revealed elevated levels of P-ATM, P-CHK2, and P-P53 in omental metastases (Fig. S9E). Our data collectively demonstrate that arginine activates the ATM/CHK2/TP53 axis through binding to DDX3X, thereby promoting DDR and metastatic progression in EOC.

### Targeting DDX3X inhibits the malignant progression of EOC

**Fig. 8:**
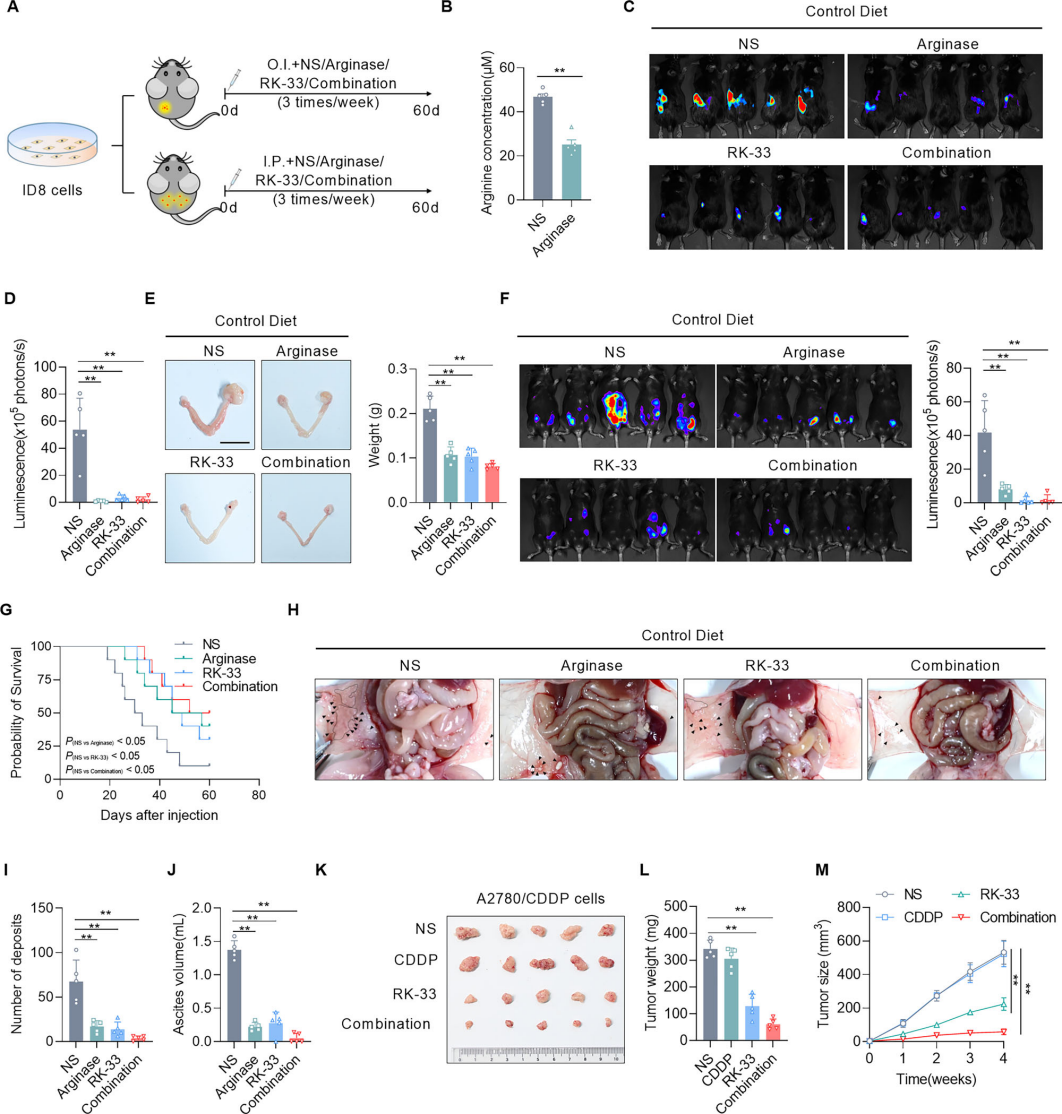
Targeting DDX3X inhibits the malignant progression of EOC. **A** Experimental scheme illustrating orthotopic ovarian cancer and intraperitoneal ovarian cancer mice with arginase (10 μg/kg, intraperitoneal injection, three times a week) or DDX3X inhibitor (RK-33, 50 mg/kg, intraperitoneal injection, three times a week) administration. **B** Plasma arginine levels in arginase-injected mice were quantified using a biochemical assay kit at 24 h post-injection. **C**, **D** Representative IVIS bioluminescence images and quantitative luminescence analysis of mice with indicated treatments at 30 days after i.o. injection of ID8-Luc cells (*n* = 5/group). **E** Representative images and weight of ovaries from orthotopic ovarian tumor mice with indicated treatments at 60 days after cell injection (*n* = 5/group). Scale bars, 1 cm. **F** Representative IVIS bioluminescence images and quantitative luminescence analysis of mice with indicated treatments at 30 days after i.p. injection of ID8-Luc cells (*n* = 5/group). **G** Kaplan–Meier analysis of mice with i.p. injection of ID8-Luc cells treated as indicated. (*n* = 10/group). **H**–**J** Representative images of peritoneal metastasis (**H**), total number of metastatic deposits (**I**), and ascites volume (**J**) in mice with indicated treatments at 35 days after i.p. injection (*n* = 5/group). **K**–**M** Representative images, tumor weight, and size of subcutaneous xenograft tumors in nude mice 30 days after injection of A2780 cisplatin-resistant cells, with the indicated treatments (*n* = 5/group). Data were shown as the mean ± SD. The *p* value was calculated using unpaired two-tailed Student’s *t*-test (**A**), two-way ANOVA (**D**–**F**, **I**, **J**, **L**), and one-way ANOVA (**M**). Survival curves were generated with the Kaplan–Meier method and analyzed using the log-rank test (**G**). *p < 0.05, **p < 0.01.

Despite the established role of arginine deprivation in inhibiting tumor growth, effectively restricting arginine in patients poses a significant clinical challenge. We therefore proposed DDX3X as a promising target to inhibit tumor progression. Arginine was depleted using arginase, and DDX3X was inhibited using its specific small-molecule inhibitor, RK-33 [31]. In vitro experiments revealed that arginase or RK-33 effectively suppressed the arginine-induced pro-tumorigenic phenotypes of EOC cells (Fig. S10A–E). Subsequently, mouse models of EOC were established via orthotopic or intraperitoneal injection of tumor cells (Fig. 8A). As expected, a significant reduction in plasma arginine level was confirmed in arginase-treated mice (Fig. 8B). The results showed that continuous arginase or RK-33 treatment inhibited the growth of orthotopic tumors, reduced the tumor burden, and decreased the proliferation of EOC cells (Fig. 8C–E and Fig. S10F, H). In addition, arginase or RK-33 treatment significantly extended the overall survival of mice with intraperitoneally metastatic EOC (Fig. 8G). The metastatic foci in the arginase or RK-33-treated mice were significantly smaller and fewer than those in the control group, and the ascites volume was largely reduced (Fig. 8F, H–J**)**. Besides, a significant reduction of Ki67+ cells treated with arginase or RK-33 provides additional support for these findings (Fig. S10G, I). Furthermore, arginase or RK-33 treatment downregulated the expression of DDR-related genes in metastatic lesions, weakening DDR and increasing DNA damage (Fig. S10J). Moreover, the combination of Arginase and RK-33 resulted in a relatively improved anti-cancer efficacy (Fig. 8C–J). Collectively, inhibition of DDX3X exhibits a tumor-suppressive effect comparable to arginine restriction, and the combination of arginase and RK-33 may represent a promising therapeutic approach for EOC.

Given the protective role of DDX3X in cisplatin-induced DNA damage, we thus investigated whether targeting DDX3X could enhance the platinum sensitivity of resistant EOC cells. The results showed that the individual administration of CDDP or RK-33 in A2780‑cis cells exhibited IC₅₀ values of 44.72 and 4.44 μM, respectively (Fig. S11A, B). And their combination demonstrated significant synergistic effects in inhibiting the proliferation of these cells, with quantitative analysis by SynergyFinder yielding mean synergy scores of 11.06 (ZIP model) and 11.04 (Bliss model) across all concentration pairs (Fig. S11C). Furthermore, combined treatment based on even half of the respective IC₅₀ values (2 μM RK‑33 + 20 μM CDDP) induced an apoptosis rate of up to 62% (Fig. S11D). Subsequently, we established a xenograft model by subcutaneously injecting CDDP-resistant A2780 cells into nude mice. The mice were then treated with CDDP, RK-33, or a combination of both. The results demonstrated that the combination treatment significantly inhibited tumor growth, leading to a reduced tumor burden (Fig. 8K–M). These findings suggest that targeting DDX3X represents a viable therapeutic strategy for sensitizing platinum-resistant EOC cells.

## Discussion

In recent years, increasing attention has been paid to the role of the metabolic reprogramming of amino acids in tumor metastasis, and an increasing number of studies have revealed that specific amino acids contribute to tumor metastasis through multiple pathways [12, 32–35]. In the present study, we discovered that metastatic EOC cells rely preferentially on exogenous arginine uptake rather than endogenous synthesis, which represents a critical metabolic adaptation to the metastatic microenvironment. Phenotypically, arginine deprivation inhibited the growth and metastases of EOC in vivo, and arginine supplementation enhanced cell proliferation, invasion, and migration in vitro. Mechanistically, arginine binds to DDX3X and promotes its nuclear retention. DDX3X then transcriptionally potentiates DDR through activating the ATM/CHK2/P53 axis, ultimately facilitating tumor progression. Notably, inhibition of DDX3X significantly suppressed tumor growth and metastasis, revealing an alternative therapeutic strategy in cases where restricting arginine uptake is challenging. In general, we identified metabolic vulnerabilities in metastatic EOC, providing a crucial theoretical basis for developing targeted treatment strategies.

Different malignancies exert diverse biological effects through metabolic reprogramming of arginine [26]. In breast cancer, TGF-β-activated macrophages consume arginine to fuel proline metabolism and collagen synthesis, thereby impairing CD8 + T cell function and weakening the response to immune checkpoint inhibitors [36]. In hepatocellular carcinoma, cancer cells form a positive feedback loop through arginine uptake and upregulation of asparagine synthase, ultimately promoting oncogenic metabolism [14]. Our study revealed that metastatic EOC presents as an arginine auxotroph, a phenotype dependent on exogenous uptake to maintain high-arginine concentration, which is also observed in other malignancies [37, 38]. Considering that there is an endogenous synthesis defect of arginine in metastatic EOC, tumor cells have to take up the exogenous arginine. Moreover, arginine functions as a crucial “signal molecule” to activate the downstream pathways, providing EOC cells with robust survival signals to cope with the genomic instability during metastasis. Therefore, EOC cells with metastasis must adapt to this metabolic stress and the survival pressure from the metastatic microenvironment by taking in a large amount of exogenous arginine. Apart from this, we also found that arginine promotes malignant phenotypes in EOC cells, while arginine deprivation suppresses tumor growth and peritoneal metastasis. Collectively, exogenous arginine plays an indispensable role in fueling the malignant progression of EOC metastases, and arginine deprivation may be a potential therapeutic strategy for EOC.

Metabolites function as sensors to couple cellular metabolic states with gene regulatory programs, integrating control at epigenetic, transcriptional, translational, and post-translational levels [39]. Excitedly, we have made an innovative discovery that arginine can directly bind to the RNA helicase DDX3X and induce its retention in the nucleus by masking its NES, which provides a new paradigm for metabolites to directly regulate the subcellular localization and function of RNA-binding proteins. Chen et al. have also found that arginine regulates the subcellular localization of specific proteins. In prostate cancer cells, arginine promotes the nuclear translocation of TEAD4 to regulate the promoters/enhancers of OXPHOS genes, thereby facilitating the transcription of OXPHOS genes [40]. To be specific, the binding of arginine may change the conformation or surface charge of the N-IDR of DDX3X, thereby affecting its interaction with the nuclear export protein CRM1, or promoting its phase separation ability to form functional transcriptional complexes in the nucleus, which is worthy of future research.

As an ATP-dependent DEAD-box family RNA helicase, DDX3X plays a pivotal role in tumor biology regarding RNA metabolic processes [41]. Additionally, DDX3X has been reported to be significantly overexpressed in EOC [16]. In this study, we found that the expression of DDX3X was significantly upregulated in omental metastasis of EOC and was associated with a poor patient prognosis. This finding extends the known association between DDX3X and EOC from primary tumors to metastatic foci, suggesting that DDX3X plays a role in regulating the metastasis of EOC. Following DNA damage, DDX3X is dynamically recruited to damage sites through a PARP1-dependent phase separation mechanism [42]. Inhibition of DDX3X leads to the accumulation of DNA double-strand breaks (DSBs) and impairs the DDR process by inhibiting non-homologous end-joining repair [31, 43]. However, the regulatory mechanism between nuclear-translocated DDX3X and the DDR pathway remains unclear. Different from the classic roles in other types of tumors, such as pancreatic cancer and breast cancer [44, 45], DDX3X has been reprogrammed in the specific context of EOC with omental metastasis. Probably, DDX3X plays a crucial bridging role in connecting the extracellular arginine nutritional status with the intracellular DDR process, as well as a vital role of an adaptive transcriptional regulatory factor that supports the proliferation and invasion of tumor cells in the stress-induced metastatic microenvironment. Our study illustrates that DDX3X undergoes nuclear retention by binding to arginine, thereby transcriptionally activating the ATM/CHK2/P53 pathway to enhance the DDR, ultimately promoting the malignant progression of EOC. These findings not only demonstrate the non-canonical function regarding transcriptional regulation of the RNA-binding protein, but also enrich the mechanisms through which the RNA-binding protein modulates the DDR process.

DDR is a vital mechanism for tumor cells to maintain genomic stability by recognizing and repairing DNA damage. Notably, dysregulation of DDR processes is common in EOC [46]. Our study revealed that omental metastases of EOC exhibit a significantly higher degree of DNA damage than the primary tumors, suggesting that detrimental factors, such as oxidative stress, may accumulate much more in the metastatic microenvironment. This finding is consistent with the prevalent observation of DNA damage in metastases across various solid tumors [47–49]. Collectively, we elucidated the mechanism by which DNA damage is positively repaired in metastatic EOC cells, thus facilitating efficient genomic stability. In parallel, Erez et al. reported that DNA damage triggers nuclear localization of ASS1, which promotes cell cycle recovery by regulating p53-associated genes [50]. These findings highlight the metabolic regulatory function of DDR pathways and establish a novel theoretical foundation for targeting DDR in EOC.

## Conclusions

In summary, the present study revealed that the omental metastases of EOC depend on the uptake of exogenous arginine, implying metabolic vulnerability in the process of metastasis. Mechanistically, arginine is actively taken up by metastatic EOC cells and binds to the RNA helicase DDX3X, which promotes the nuclear retention of DDX3X. The nuclear DDX3X enhanced DDR through activating the ATM/CHK2/P53 axis. Therefore, arginine-DDX3X signaling not only plays a crucial role in promoting EOC cell survival and metastasis, but also essentially represents an adaptive reprogramming of EOC cells in response to microenvironmental metabolic stress. Furthermore, arginine restriction and targeting DDX3X may be a feasible strategy for preventing and treating EOC metastasis, and more potential combination therapy strategies should be investigated in the future.

## Supplementary information

Supplemental figures and tables

Supplemental data - western blot

## Data Availability

The RNA-seq supporting the findings of the present study has been deposited at GSA-Human under accession code PRJCA039923. All other supporting data of this study are available from the corresponding author on reasonable request.
