# Supplementary material for: Arginine dependency in omental metastasis of epithelial ovarian cancer reveals a therapeutic vulnerability
Source: Cell Death Dis. 2026 Mar 24;17(1):354. doi: 10.1038/s41419-026-08606-3 (PMC13039504; doi:10.1038/s41419-026-08606-3)
Supplement: Supplementary file 1 — Supplemental figures and tables [file 41419_2026_8606_MOESM1_ESM.docx]

**Supplemental Figures**

**Fig. S1.**


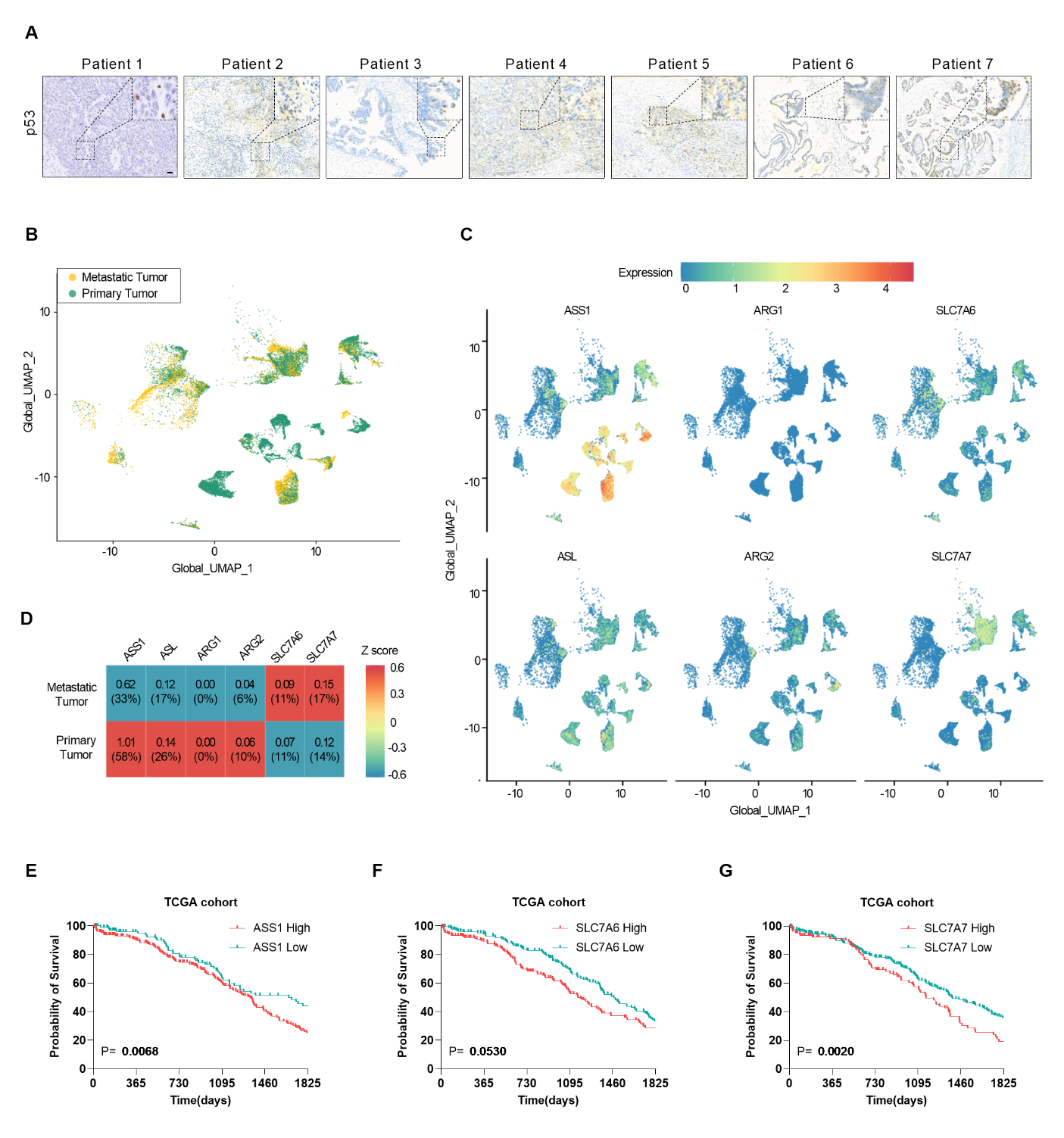


**Fig. S1.**

**(A)** Immunohistochemistry (IHC) for TP53 was performed to confirm the wild-type status of tumors from the seven EOC patients. Scale bars, 40 μm.

**(B)** UMAP plots depicting metastatic and primary tumor cells in high-grade serous ovarian carcinoma patients.

**(C-D)** UMAP plots and heat map showing expression levels of ASS1, ASL, ARG1, ARG2, SLC7A6, and SLC7A7 in metastatic and primary tumor cells.

**(E)** Kaplan–Meier analysis of overall survival in TCGA-OV patients stratified by ASS1 expression (high: n=243, low: n=106).

**(F)** Kaplan–Meier analysis of overall survival in TCGA-OV patients stratified by SLC7A6 expression (high: n=177, low: n=172).

**(G)** Kaplan–Meier analysis of overall survival in TCGA-OV patients stratified by SLC7A7 expression (high: n=108, low: n=241).

Survival curves were generated with the Kaplan–Meier method and analyzed using the log-rank test (E-G).*p<0.05, **p<0.01.

**Fig. S2.**


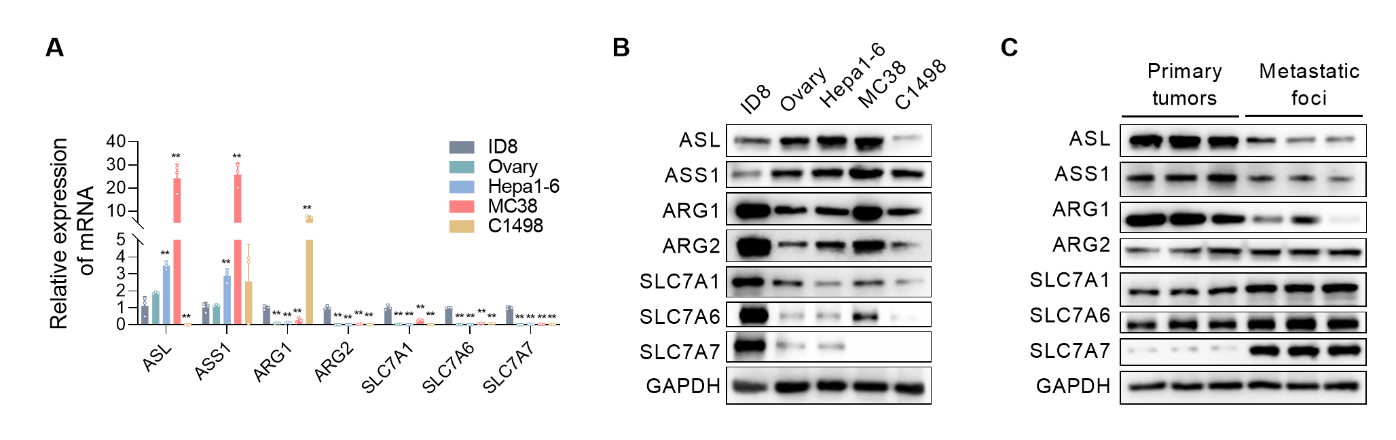


**(A)** Quantitative Real-time polymerase chain reaction (RT-qPCR) analyses of ASS1, ASL, ARG1, ARG2, SLC7A1, SLC7A6, and SLC7A7 mRNA levels in ID8 cells, mouse normal ovarian tissue, mouse liver cancer cells Hep1-6, mouse intestinal cancer cells MC38, and mouse leukemia cells C1498 cells.

**(B)** Western blot analyses of ASS1, ASL, ARG1, ARG2, SLC7A1, SLC7A6, SLC7A7, and GAPDH protein levels in ID8 cells, mouse normal ovarian tissue, mouse liver cancer cells Hep1-6, mouse intestinal cancer cells MC38, and mouse leukemia cells C1498 cells.

**(C)** Western blot analyses of ASS1, ASL, ARG1, ARG2, SLC7A1, SLC7A6, SLC7A7, and GAPDH protein levels in primary tumor and metastatic foci derived from ID8 cells.

Data are shown as the mean±SD. The p-value was calculated using one-way ANOVA (A). *p<0.05, **p<0.01.

**Fig. S3.**


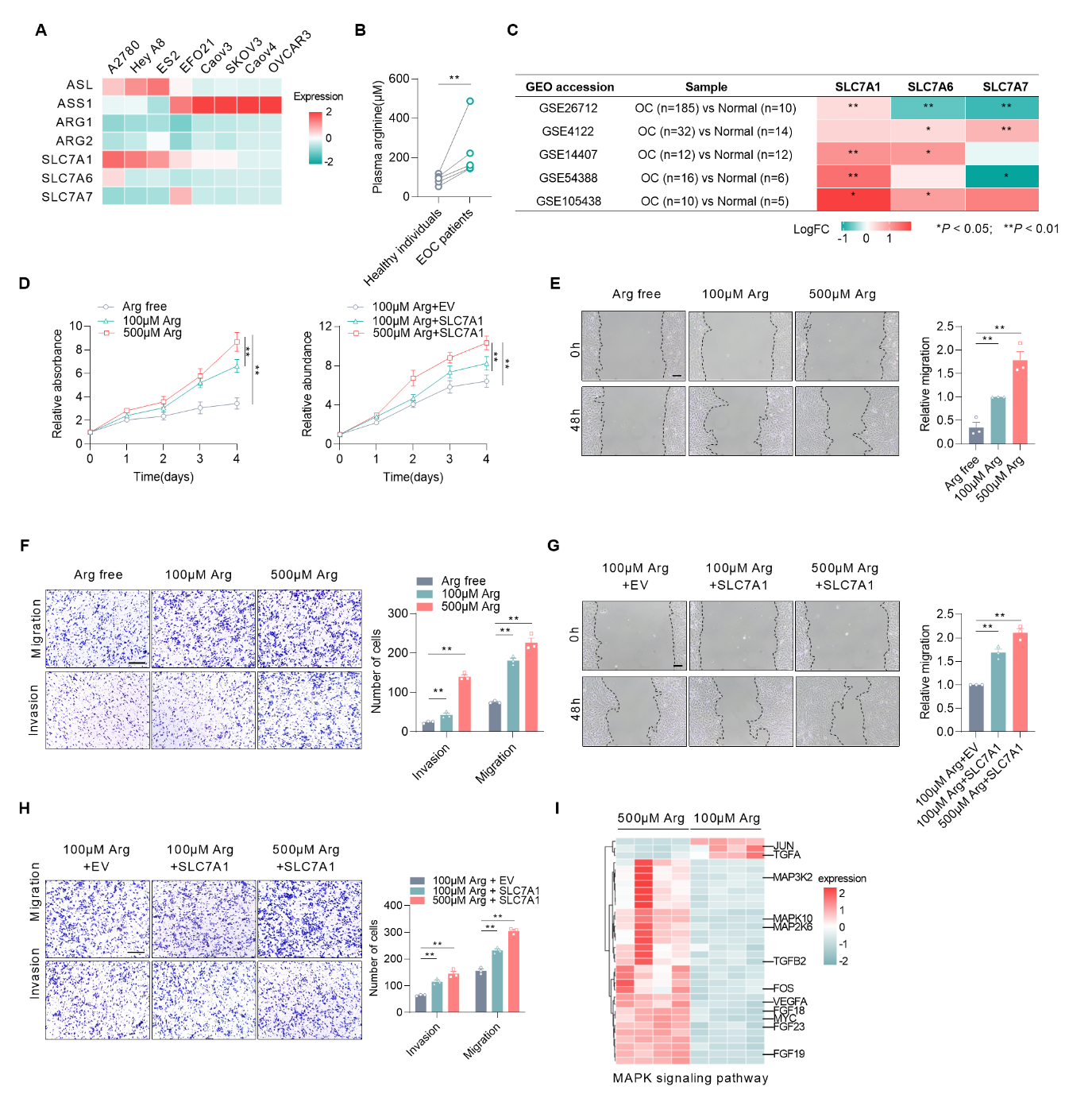


**Fig. S3.**

**(A)** The heatmap displays the mRNA expression levels of ASS1, ASL, SLC7A1, SLC7A6, and SLC7A7 in epithelial ovarian cancer cell lines, as analyzed using data from the Human Protein Atlas (HPA) database.

**(B)** Scatter plots show the abundance of arginine in the peripheral blood from EOC patients with omental metastases and age-matched healthy individuals (n=5).

**(C)** Log_2_ Fold change in arginine transporter SLC7A1 gene expression between normal ovary and ovarian cancer.

**(D)** Cell proliferation in Hey A8 cells treated with indicated treatment was assessed by CCK-8 assay.

**(E)** Representative images of wound-healing assays in Hey A8 cells with treatment as indicated. Scale bars: 100 μm.

**(F)** Transwell migration and invasion assays of Hey A8 cells with treatment as indicated. Scale bars: 100 μm.

**(G)** Representative images of wound-healing assay for Hey A8 cells with treatment as indicated. Scale bars: 100 μm.

**(H)** Transwell migration and invasion assays of Hey A8 cells with treatment as indicated. Scale bars: 100 μm.

**(I)** Heatmap of gene expression levels involving MAPK signaling pathway in A2780 cells treated with 100μM and 500μM arginine (n=4) by RNA-seq.

Data are shown as the mean±SEM. The p-value was calculated using paired Student’s t-test (A), two-way ANOVA (D), one-way ANOVA (E-H). *p<0.05, **p<0.01.

**Fig. S4.** **
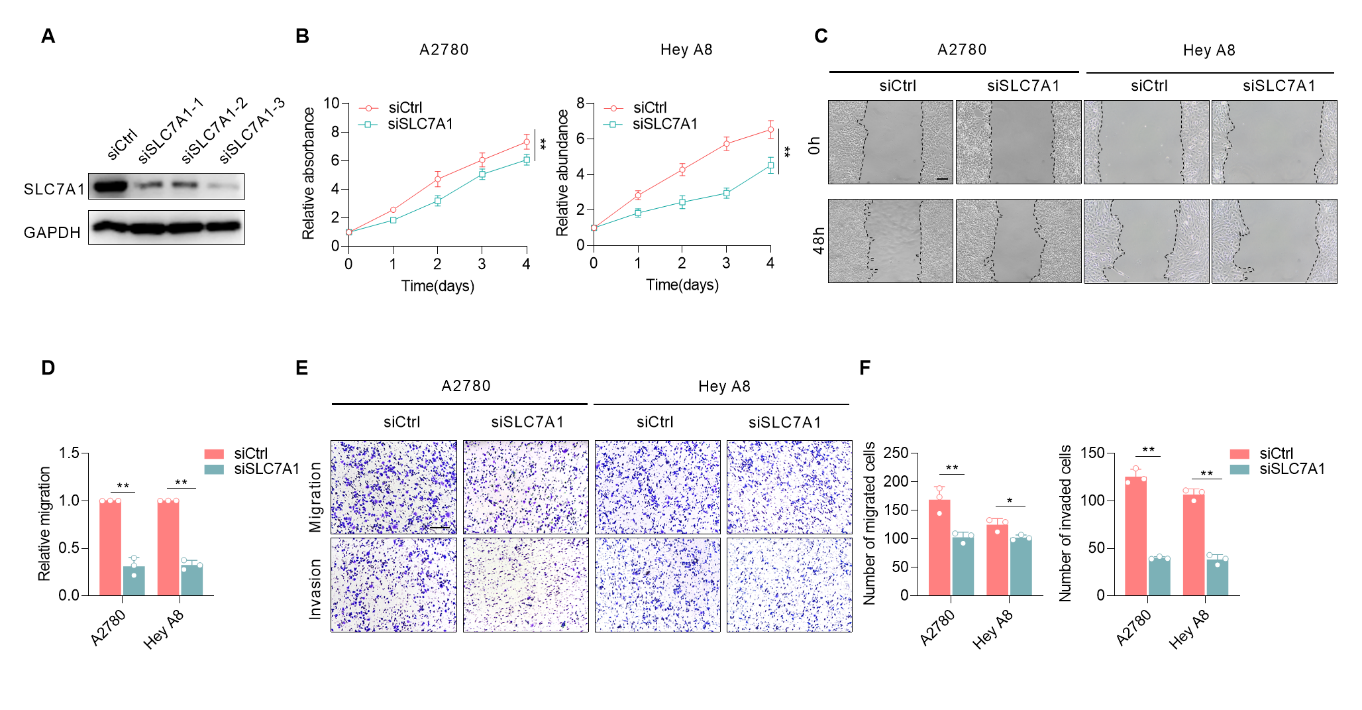
**

**(A)** Western blot analysis of SLC7A1 and GAPDH protein levels in A2780 cells transfected with siRNA targeting SLC7A1 or control siRNA.

**(B)** Cell proliferation assessed by CCK-8 assay in A2780 and Hey A8 cells with indicated treatment.

**(C-D)** Representative images of wound-healing assay for A2780 and Hey A8 cells with treatment as indicated. Scale bars: 100 μm.

**(E-F)** Transwell migration and invasion assays for A2780 and Hey A8 cells with treatment as indicated. Scale bars: 100 μm.

Data are shown as the mean±SEM. The p-value was calculated using two-way ANOVA (B),one-way ANOVA (F), and unpaired two-tailed Student’s t-test (D). *p<0.05, **p<0.01.

**Fig. S5.**


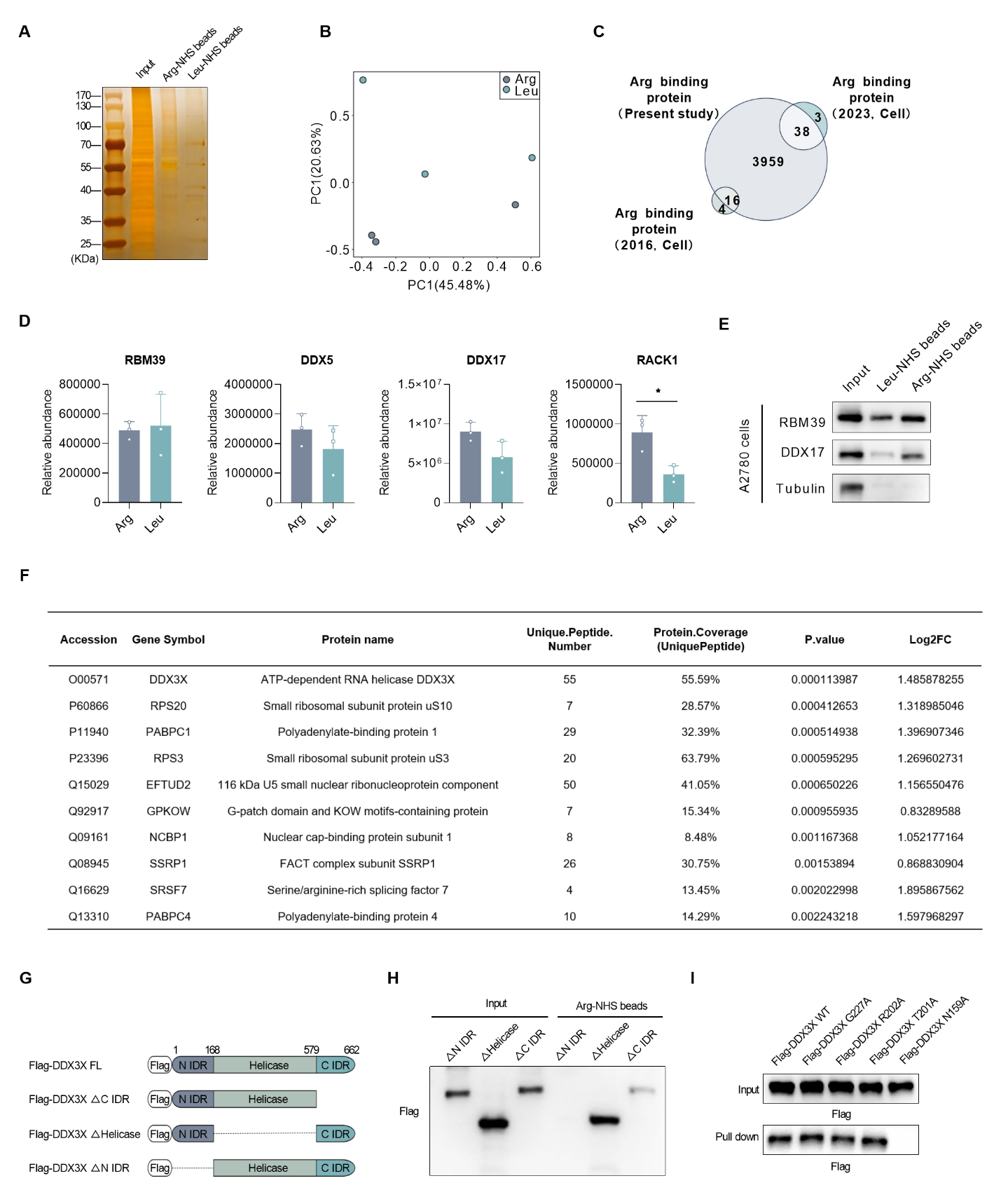


**Fig. S5.**

**(A)** Silver staining analysis of the protein enriched by L-leucine- and L-arginine-immobilized NHS magnetic beads.

**(B)** Principal component analysis of proteins pulled down using L-leucine- or L-arginine-immobilized NHS magnetic beads.

**(C)** Venn diagram comparing arginine-binding proteins identified in this study versus prior studies.

**(D)** Relative abundance of RBM39, DDX5, DDX17 and RACK1 protein captured by arginine or leucine-coupled magnetic beads.

**(E)** Western blot detection of DDX17 and RBM39 in A2780 cells with arginine treatment, and elution after purification with L-leucine- or L-arginine-immobilized NHS magnetic beads.

**(F)** Top 10 RNA-binding proteins among arginine-interacting proteins.

**(G)** Schematic diagram of Flag-DDX3X truncations.

**(H)** Western blot detection of Flag-tagged proteins in lysates from A2780 cells transfected with different DDX3X truncations, purified using arginine-immobilized NHS magnetic beads.

**(I)** Western blot detection of Flag-tagged proteins in lysates from A2780 cells transfected with different DDX3X mutants, purified using arginine-immobilized NHS magnetic beads.

Data are shown as the mean±SD. The p-value was calculated using unpaired two-tailed Student’s t-test (D). *p<0.05.

**Fig. S6.**


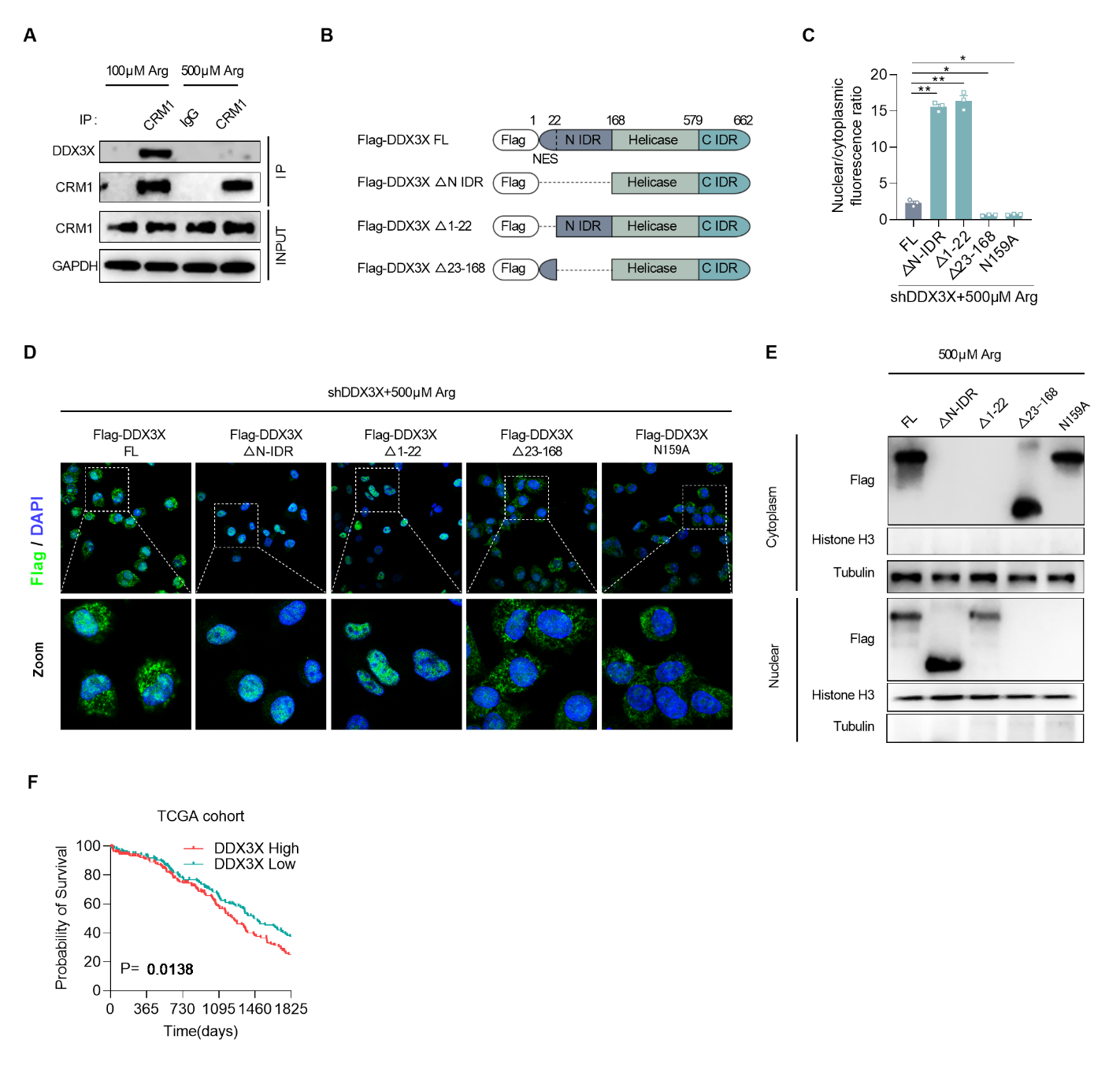


**Fig. S6.**

**(A)** Western blot analysis of CRM1 immunoprecipitates and corresponding whole-cell lysates (input) from A2780 cells following treatment with 100 μM or 500 μM arginine.

**(B)** Schematic diagram of Flag-DDX3X truncations.

**(C)** Quantification of the nuclear-to-cytoplasmic ratio of Flag fluorescence intensity in A2780 shDDX3X cells treated with arginine as indicated. Scale bars, 10 μm.

**(D)** Representative confocal microscopy images of FLAG immunofluorescence in A2780 shDDX3X cells treated with arginine as indicated. Scale bars, 10 μm.

**(E)** Western blot analyses of Flag, histone H3, and tubulin protein levels in the cytoplasmic and nuclear fractions of A2780 shDDX3X cells under the indicated treatments.

**(F)** Kaplan–Meier curves showing overall survival of OC patients with high (n = 196) vs. low (n = 153) DDX3X levels in TCGA-OV cohort.

Data are shown as the mean ± SEM. The p-value was calculated using one-way ANOVA (C). Survival curves were generated with the Kaplan–Meier method and analyzed using the log-rank test (F). *p<0.05, **p<0.01.

**Fig. S7.**


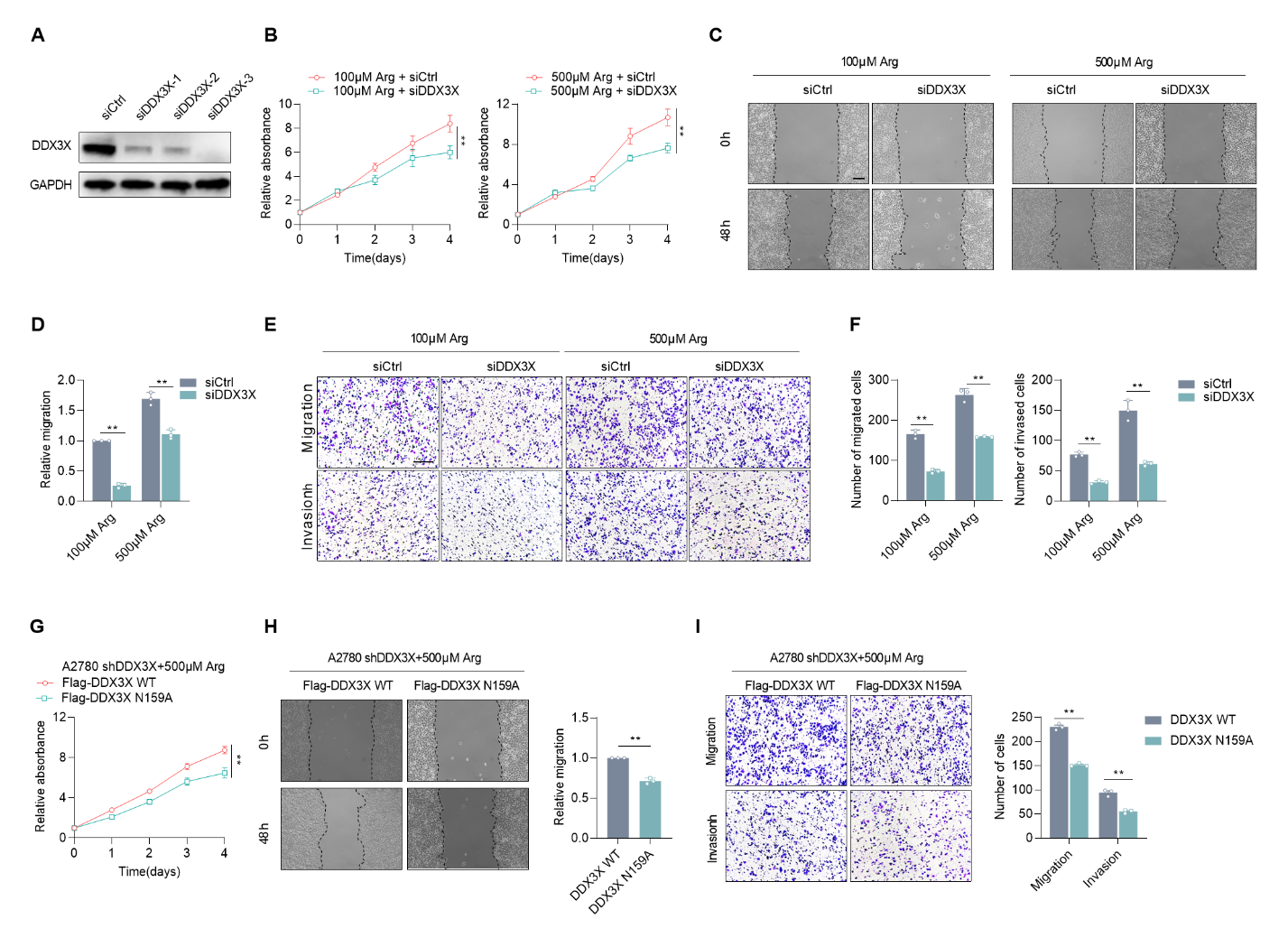


**Fig. S7.**

**(A)** Western blot analysis of DDX3X and GAPDH protein levels in A2780 cells transfected with siRNA targeting DDX3X or control siRNA.

**(B)** Cell proliferation in A2780 cells with indicated treatment was assessed by CCK-8 assay.

**(C-D)** Representative images of wound-healing assays in A2780 cells treated with si-DDX3X. Scale bars: 100 μm.

**(E-F)** Transwell migration and invasion assays for A2780 cells with treatment as indicated. Scale bars: 100 μm.

**(G)** Cell proliferation was assessed by CCK-8 assay in stable DDX3X-knockdown A2780 cells following transfection with the indicated DDX3X constructs and treatment with 500 μM arginine.

**(H)** Representative images of wound-healing assays in stable DDX3X-knockdown A2780 cells transfected with the indicated DDX3X construct and treated with 500 μM arginine. Scale bars: 100 μm.

**(I)** Transwell migration and invasion assays for in stable DDX3X-knockdown A2780 cells transfected with the indicated DDX3X construct and treated with 500 μM arginine. Scale bars: 100 μm.

Data are shown as the mean±SEM. The p-value was calculated using two-way ANOVA (B and G), and unpaired two-tailed Student’s t-test (D, F,H, and I). *p<0.05, **p<0.01.

**Fig. S8.**


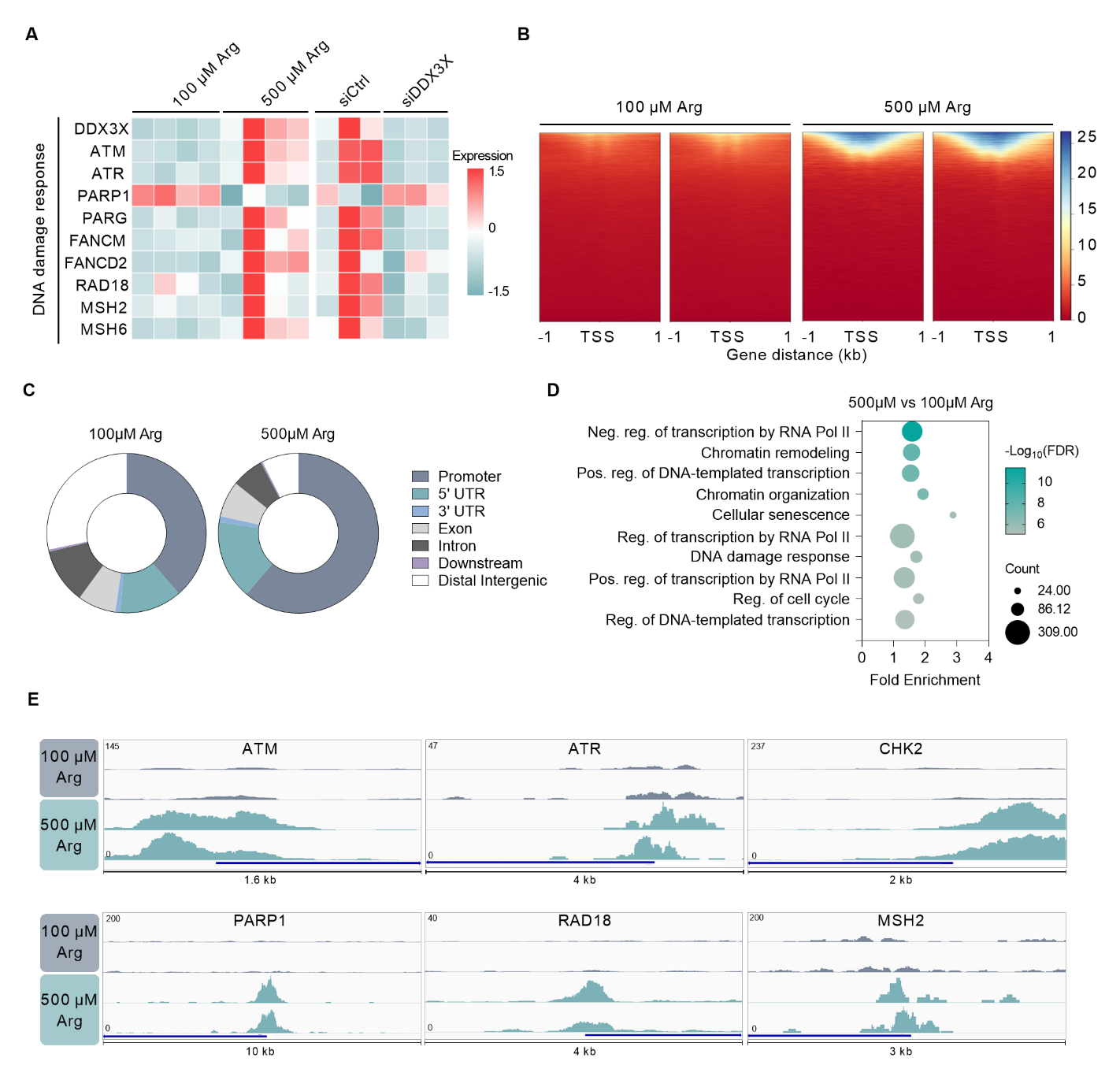


**Fig. S8.**

**(A)** Heatmap based on RNA-seq showing the DDR genes between A2780 cells with indicated treatments.

**(B)** CUT&Tag signal density heatmaps for A2780 cells treated with 100 μM or 500 μM arginine, ranked by DDX3X read intensity within ±1 kb of transcription start sites (TSSs).

**(C)** The genomic distribution of DDX3X binding peaks across various functional elements of genes was analyzed in A2780 cells treated with 100 μM or 500 μM arginine.

**(D)** KEGG pathway enrichment analysis was performed on genes corresponding to the differential binding peaks.

**(E)** The peaks map depicts DDX3X binding sites at DDR gene loci(ATM, ATR, CHK2, PARP1, RAD18, MSH2) in A2780 cells following treatment with 100 μM or 500 μM arginine.

**Fig. S9.**

**
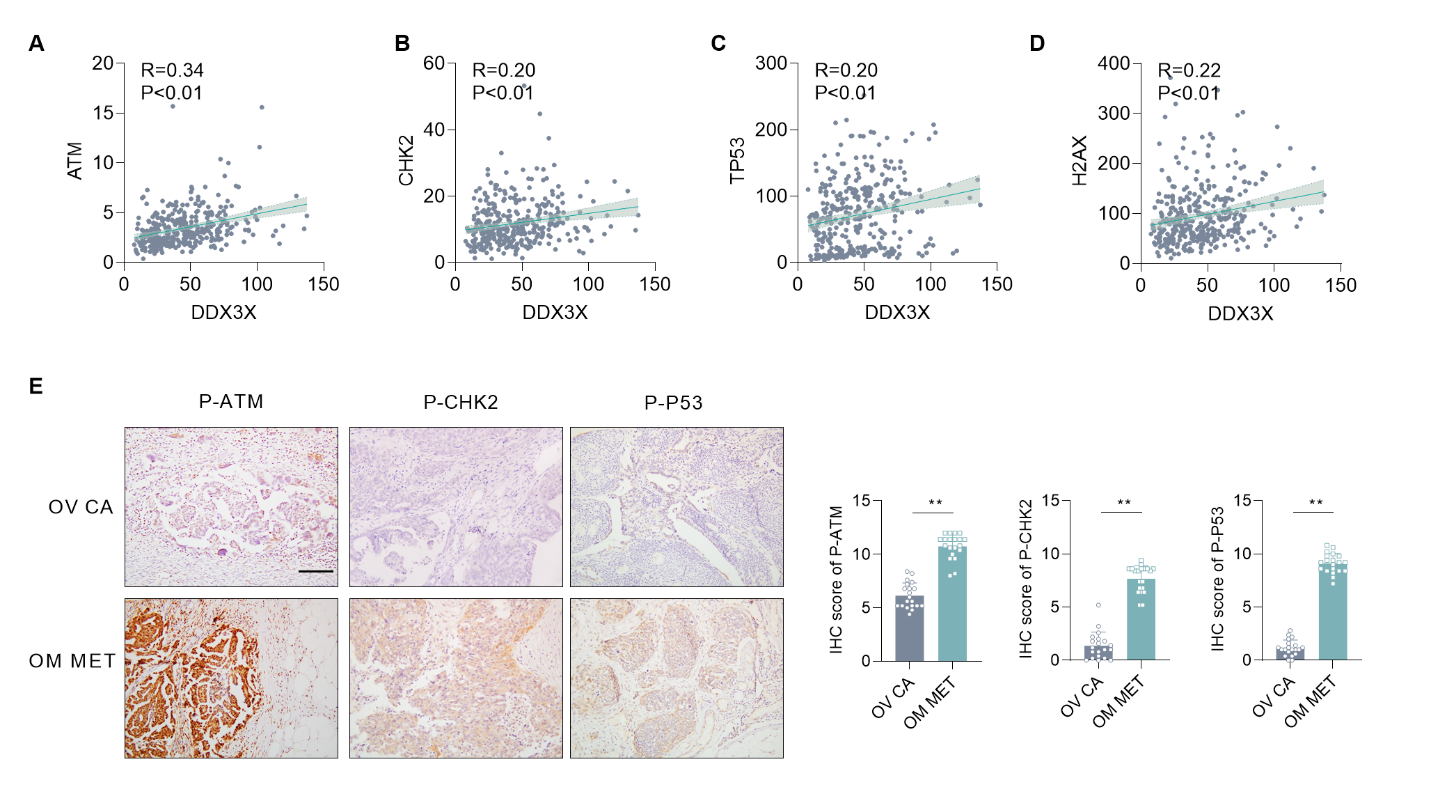
**

**(A-D)** Correlation analysis between ATM, CHK2, TP53, H2AX and DDX3X in the TCGA-OV dataset.

**(E)** Representative image and quantification of immunohistochemical staining for P-ATM, P-CHK2, and P-P53 in primary tumors and omentum metastasis (n=20). Scale bars, 100 μm.

Data are shown as the mean±SEM. The correlation coefficients and p values are from Pearson’s correlation test (A-D). The p-value was calculated using unpaired two-tailed Student’s t-test (E). **p<0.01.

**Fig. S10.**

**
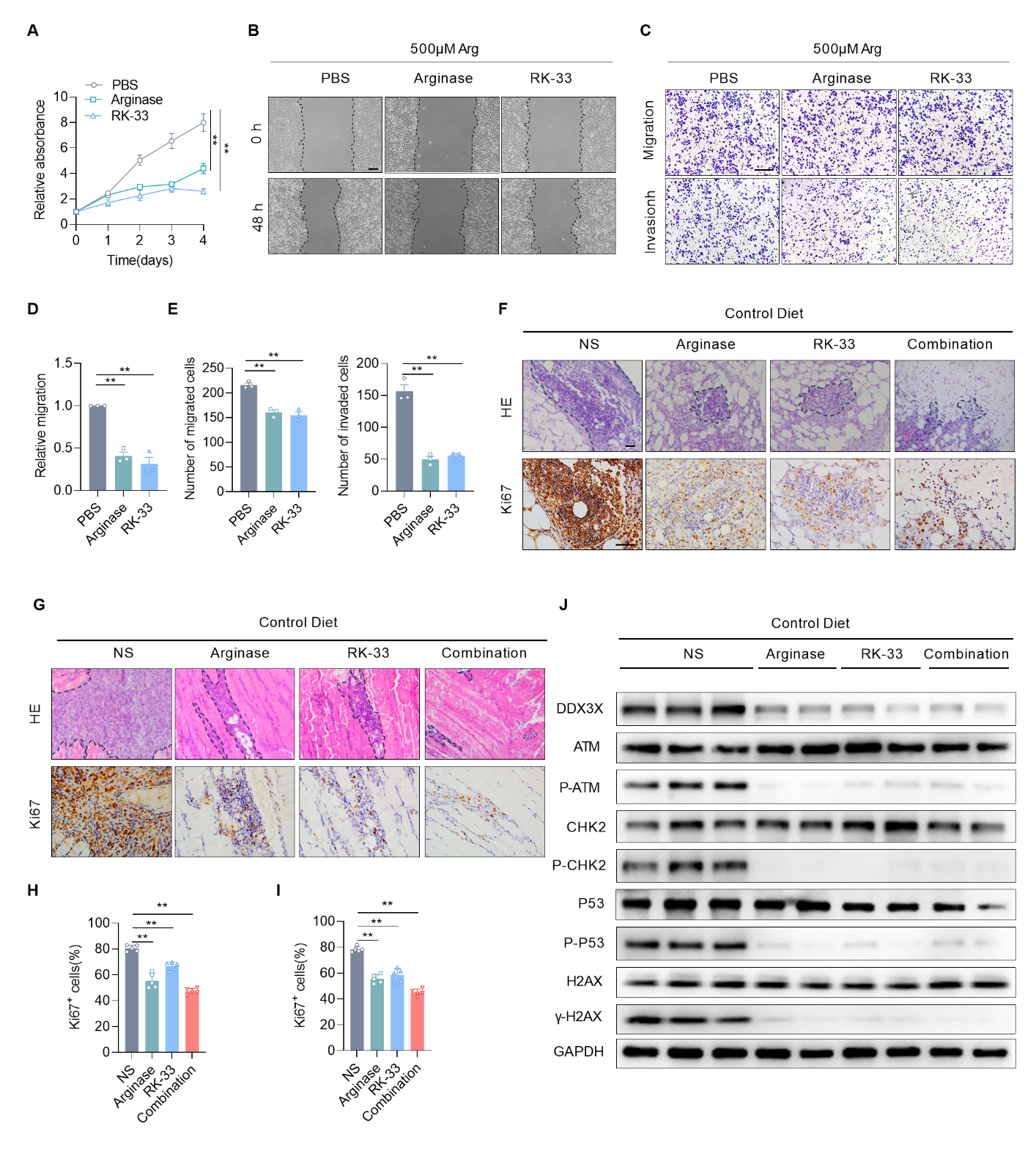
**

**Fig. S10.**

**(A)** Cell proliferation assessed by CCK-8 assay in A2780 cells with indicated treatments.

**(B-E)** Representative images and quantification of wound-healing, transwell migration, and invasion assays for A2780 cells with treatments as indicated. Scale bars: 100 μm.

**(F)** Representative images of H&E and Ki-67 staining in mouse ovarian tumors (n = 5/group). Scale bars, 50 μm.

**(G)** Representative images of H&E and Ki-67 staining in mouse peritoneal metastases (n = 5/group). Scale bars, 50 μm.

**(H)** Percentage of Ki67-positive cells in orthotopic tumors of mice with indicated treatments (n=5/group).

**(I)** Percentage of Ki67-positive cells of tumors from the intraperitoneal ovarian cancer model with indicated treatments (n=5/group).

**(J)** Western blot analysis of DDX3X, ATM, Phospho-ATM (Ser 1981), CHK2, Phospho-CHK2 (Thr68), P53, Phospho-P53 (Ser15), H2AX, γH2AX and GAPDH protein levels in peritoneal metastases from mice with indicated treatment.

Data are shown as the mean±SEM. The p-value was calculated using two-way ANOVA (A), one-way ANOVA (D, E, H, and I). *p<0.05, **p<0.01.

**Fig. S11.**


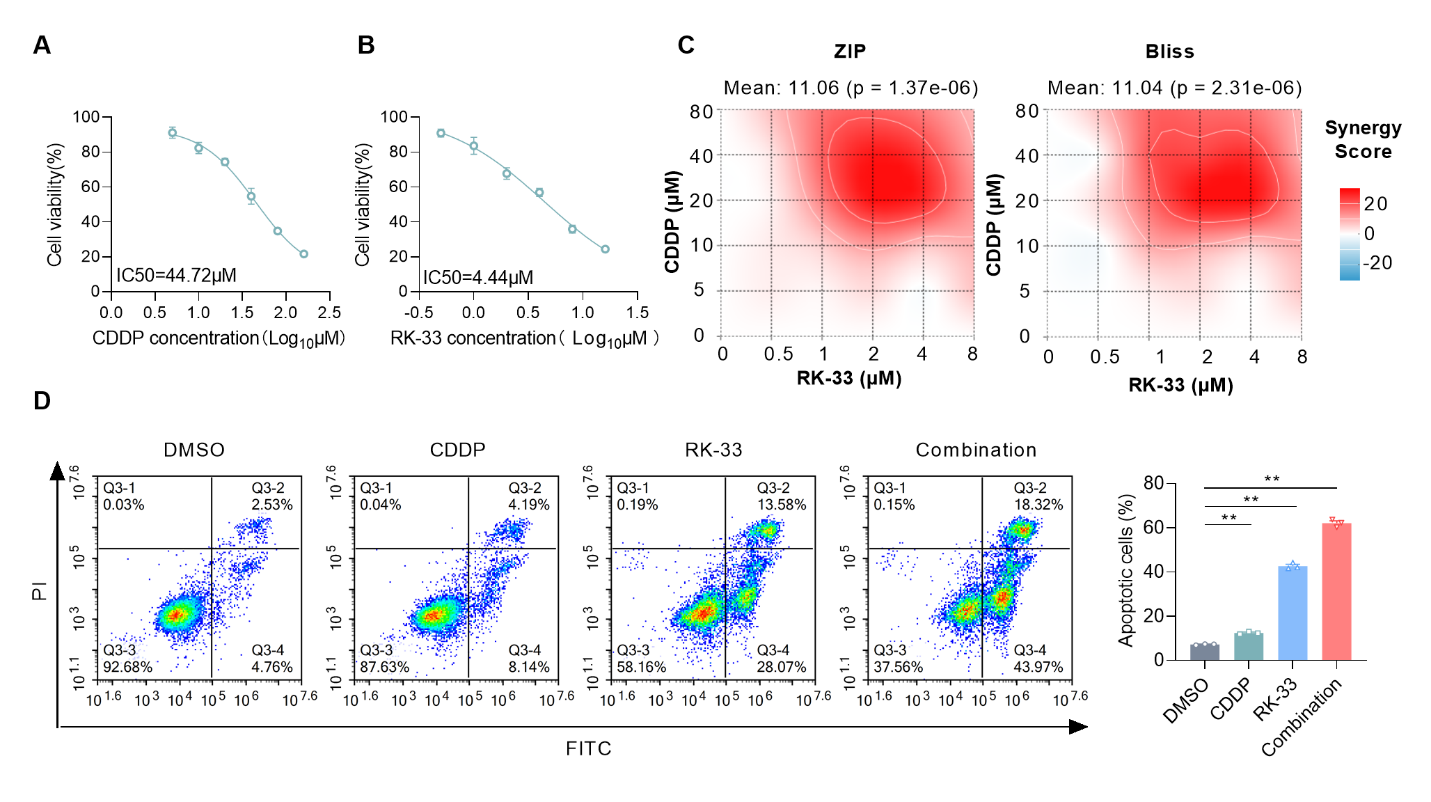


**(A)** The suppression rate of CDDP on the proliferation of A2780-cis cells as determined by CCK8 assay.

**(B)** The suppression rate of RK-33 on the proliferation of A2780-cis cells as determined by CCK8 assay.

**(C)** The viability of A2780 cisplatin-resistant cells treated with either CDDP or RK-33 alone or in combination at 48 h. Cell viability was determined by CCK- 8 assay. Synergy was assessed by the Synergy Finder website (https://synergyfinder.org).

**(D)** Apoptosis of A2780 cisplatin-resistant cells after treatment with DMSO, CDDP (20 μM), RK-33 (2 μM) and the combination.

Data are shown as the mean±SD. Dose-response curves were generated by plotting the inhibition rate against the logarithm of drug concentration. The half-maximal inhibitory concentration (IC₅₀) was calculated by fitting the data to a four-parameter logistic (4PL) nonlinear regression model using GraphPad Prism 9.0.0. The p-value was calculated using one-way ANOVA (D). *p<0.05, **p<0.01.

Supplemental Tables

**Table. S1. Sequence of siRNA.**

| **siRNA** | **Sequence** | |
| --- | --- | --- |
| siSLC7A1-1 | sense | CAGCUUACCUCUACAGCUA |
|  | antisense | UAGCUGUAGAGGUAAGCUG |
| siSLC7A1-2 | sense | CAUCGGUACUUCAAGCGUA |
|  | antisense | UACGCUUGAAGUACCGAUG |
| siSLC7A1-3 | sense | CCAAGACGGGCUCAGCUUA |
|  | antisense | UAAGCUGAGCCCGUCUUGG |
| siDDX3X-1 | sense | GGACAUUUCAAAUGUGAAA |
|  | antisense | UUUCACAUUUGAAAUGUCC |
| siDDX3X-2 | sense | GGGUGGAAGAAUCAGACAA |
|  | antisense | UUGUCUGAUUCUUCCACCC |
| siDDX3X-3 | sense | GGUGUUAGAUGAAGCUGAU |
|  | antisense | AUCAGCUUCAUCUAACACC |

**Table. S2. Antibodies used in western blot, immunofluorescence, and IHC.**

| **Antibody** | **Company** | **Cat lot** | **Host** | **Working concentration** |
| --- | --- | --- | --- | --- |
| ASS1 | Proteintech | 66036-1-Ig | Mouse | IHC 1:200 |
| ARG1 | Proteintech | 66129-1-Ig | Mouse | IHC 1:200 |
| SLC7A6 | Proteintech | 13823-1-AP | Rabbit | IHC 1:200 |
| SLC7A1 | Proteintech | 14195-1-AP | Rabbit | WB 1:1000 |
| DDX3X | Proteintech | 11115-1-AP | Rabbit | IHC 1:200 WB 1:1000 |
| ATM | Selleck | F0216 | Rabbit | WB 1:1000 |
| Phospho-ATM (Ser1981) | Cell signaling technology | #5883 | Rabbit | WB 1:1000 |
| Phospho-ATM (Ser1981) | Abcam | ab81292 | Rabbit | IHC 1:200 |
| ATR | Selleck | F0773 | Rabbit | WB 1:1000 |
| Phospho-ATR (Ser428) | Cell signaling technology | #2853 | Rabbit | WB 1:1000 |
| CHK2 | Cell signaling technology | #2662 | Rabbit | WB 1:1000 |
| Phospho-Chk2 (Thr68) | Selleck | F0243 | Rabbit | WB 1:1000 IHC 1:200 |
| P53 | Cell signaling technology | # 2524 | Mouse | WB 1:1000 |
| Phospho-P53(S15) | Abcam | ab278683 | Rabbit | WB 1:1000 |
| Phospho-P53(S15) | Proteintech | 28961-1-AP | Rabbit | IHC 1:100 |
| H2AX | Selleck | F0284 | Rabbit | WB 1:1000 |
| γH2AX | Beyotime | AF5836 | Rabbit | IHC 1:100 IF 1:50  WB 1:1000 |
| Histone H3 | Selleck | F0057 | Rabbit | WB 1:1000 |
| Flag | Proteintech | 20543-1-AP | Rabbit | WB 1:1000 |
| Ki67 | Abcam | ab16667 | Rabbit | IHC 1:200 |
| GAPDH | Proteintech | 10494-1-AP | Rabbit | WB 1:1000 |
| RBM39 | Proteintech | 21339-1-AP | Rabbit | WB 1:1000 |
| DDX17 | Proteintech | 19910-1-AP | Rabbit | WB 1:1000 |
| Tubulin | Proteintech | 11224-1-AP | Rabbit | WB 1:1000 |
| HRP-conjugated Goat Anti-Mouse IgG(H+L) | Proteintech | SA00001-1 | Goat | 1:10000 |
| HRP-conjugated Goat Anti-Rabbit IgG(H+L) | Proteintech | SA00001-2 | Goat | 1:10000 |
| CoraLite488-conjugated Goat Anti-Mouse IgG(H+L) | Proteintech | SA00013-1 | Goat | 1:200 |
| CoraLite488-conjugated Goat Anti-Rabbit IgG(H+L) | Proteintech | SA00013-2 | Goat | 1:200 |
| CRM1 | Proteintech | 27917-1-AP | Rabbit | WB 1:2000 IP 1:50 |

**Table. S3. qPCR primer information.**

| Gene | Sequence(5’-3’) | |
| --- | --- | --- |
| mASS1 | Forward | ACACCTCCTGCATCCTCGT |
|  | Reverse | GCTCACATCCTCAATGAACACCT |
| mASL | Forward | CTATGACCGGCATCTGTGGAA |
|  | Reverse | AGCAACCTTGTCCAACCCTTG |
| mSLC7A1 | Forward | CTGCCTCAACACCTATGACCT |
|  | Reverse | GAGAGCAGCAATCAAGAAGGAG |
| mSLC7A6 | Forward | GCCTGCGTATGTCTGCTGA |
|  | Reverse | GCCCATGATAATGATGGCAATGA |
| mSLC7A7 | Forward | AGCACCAAGTATGAAGTGGCT |
|  | Reverse | ACACGCCATTAAGCAGGGAG |
| mARG1 | Forward | CTCCAAGCCAAAGTCCTTAGAG |
|  | Reverse | AGGAGCTGTCATTAGGGACATC |
| mARG2 | Forward | TCCTCCACGGGCAAATTCC |
|  | Reverse | GCTGGACCATATTCCACTCCTA |
| mGAPDH | Forward | TGGCCTTCCGTGTTCCTAC |
|  | Reverse | GAGTTGCTGTTGAAGTCGCA |
| hSLC7A1 | Forward | ATCATCGGTACTTCAAGCGTAGC |
|  | Reverse | GGCGTTCAGAGTCATGTGTGT |
| hGAPDH | Forward | GGAGCGAGATCCCTCCAAAAT |
|  | Reverse | GGCTGTTGTCATACTTCTCATGG |
