# Supplementary material for: Arginine dependency in omental metastasis of epithelial ovarian cancer reveals a therapeutic vulnerability
Source: Cell Death Dis. 2026 Mar 24;17(1):354. doi: 10.1038/s41419-026-08606-3 (PMC13039504; doi:10.1038/s41419-026-08606-3)

Western blots - original data

PageRuler Prestained Protein Ladder (Thermo Scientific #26617) were used in WB detection.

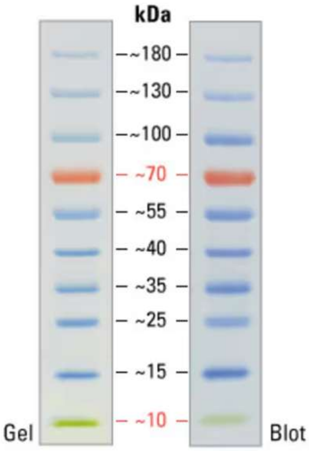

Figure 1H

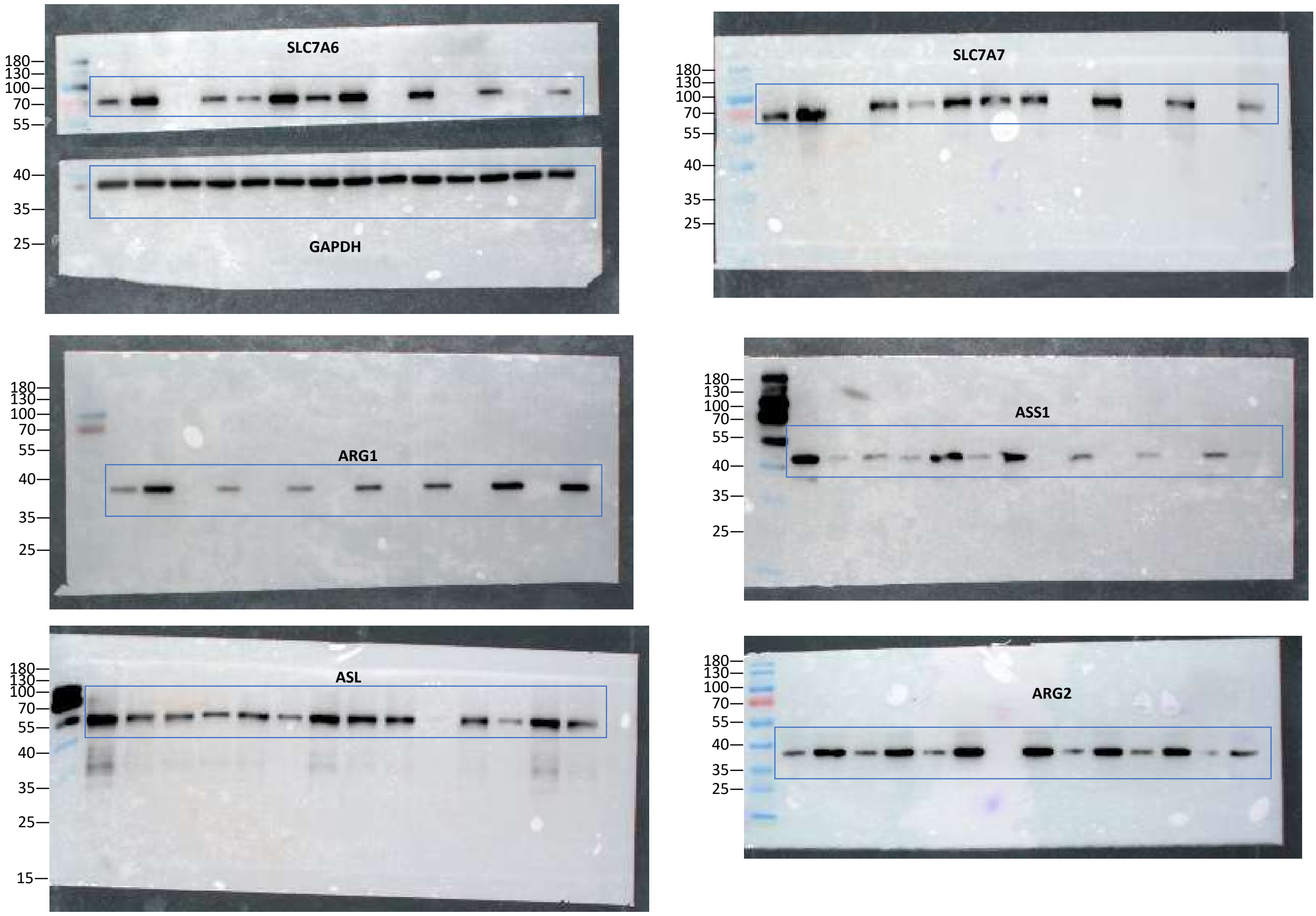

Figure 3C

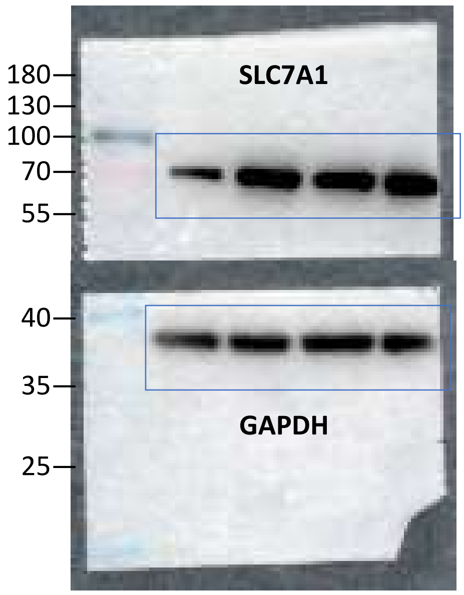

Figure 4G

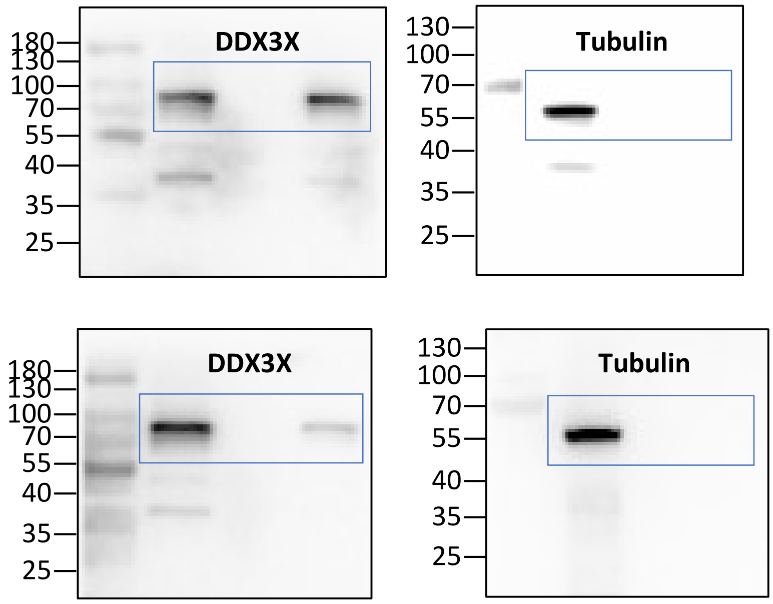

Figure 5B

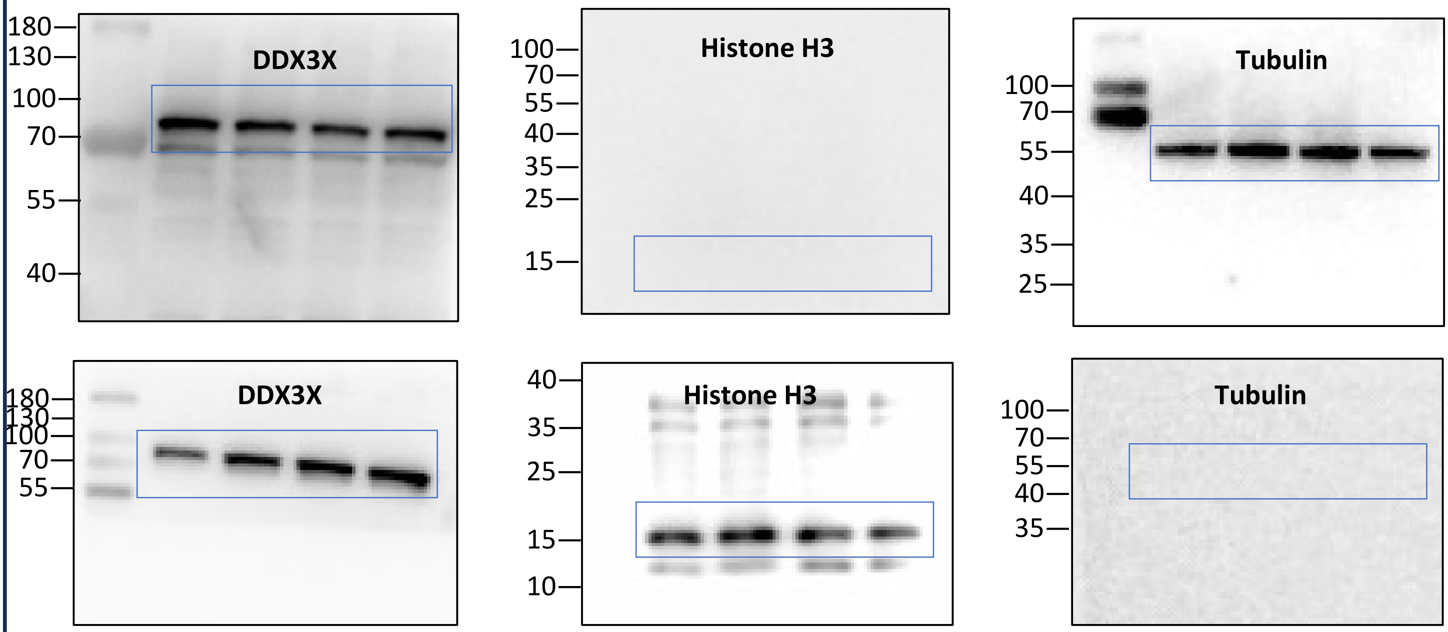

Figure 5D

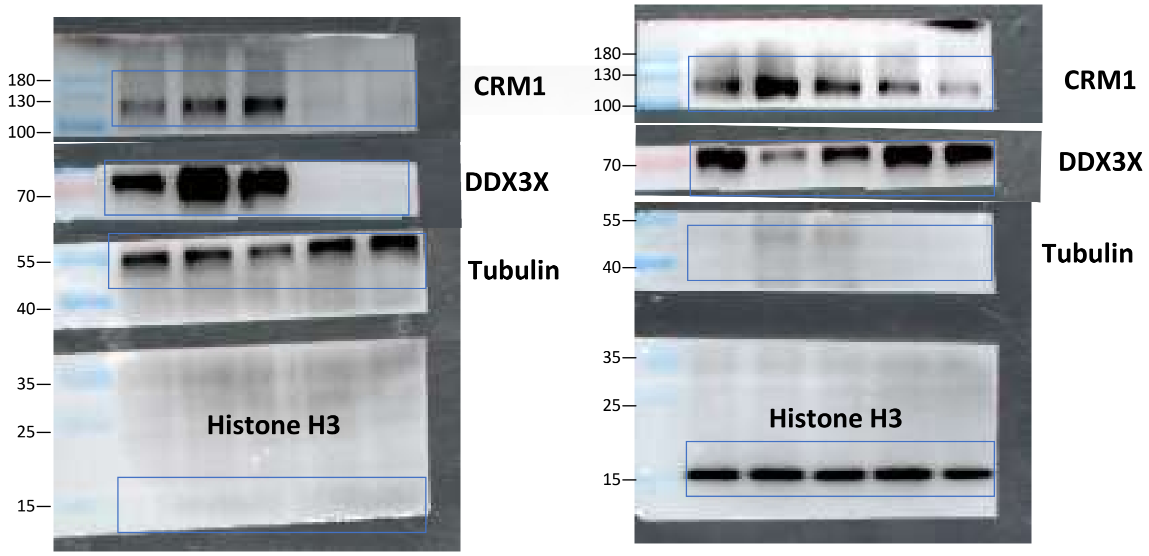

Figure 5F

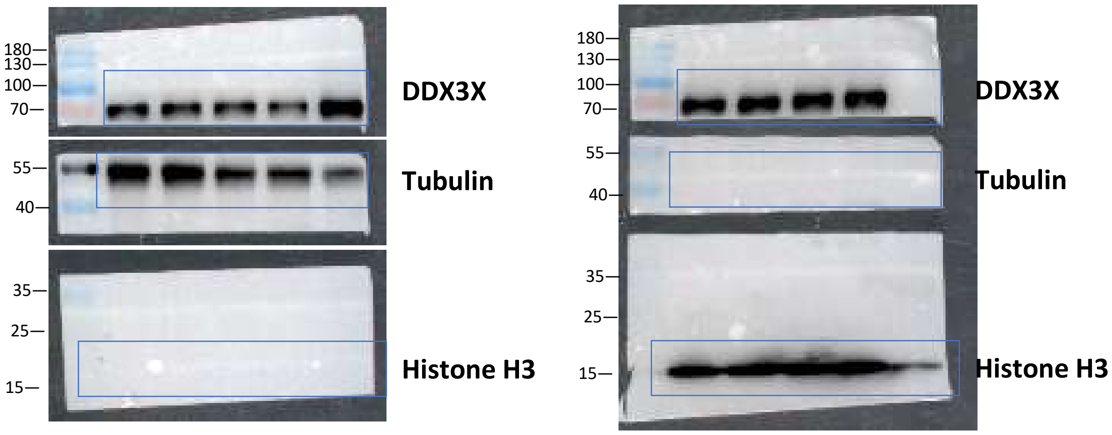

Figure 6D

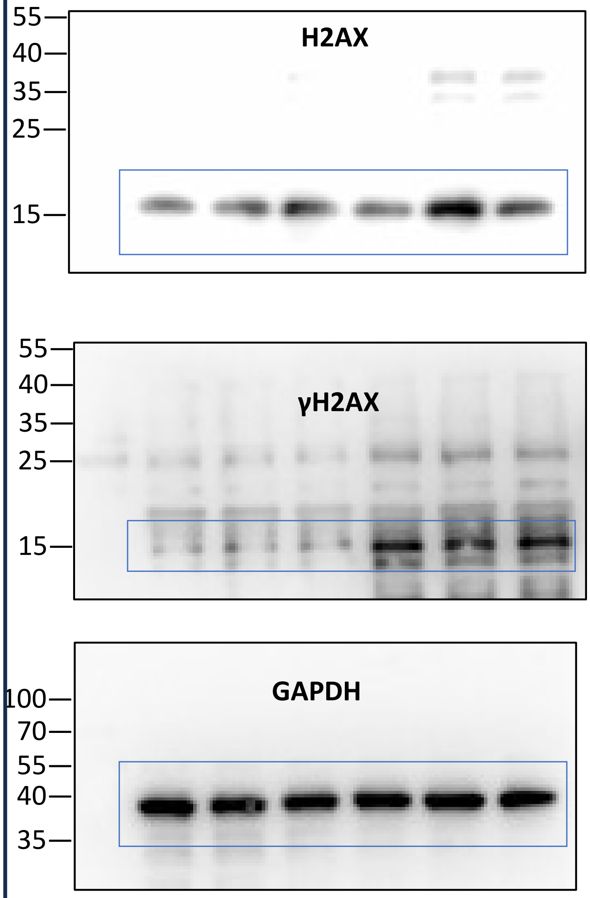

Figure 7B

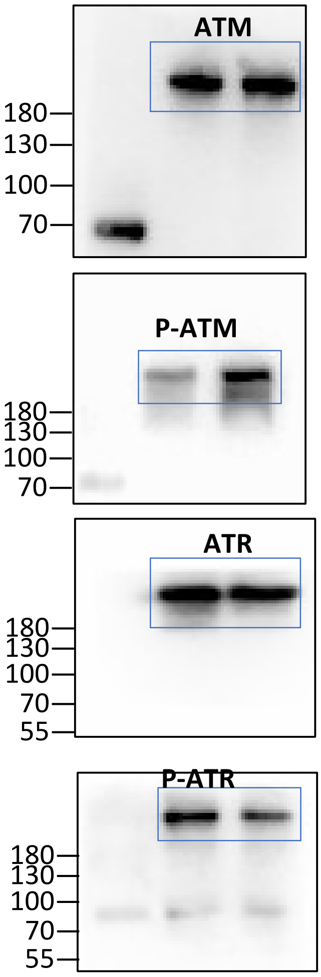

Figure 7C

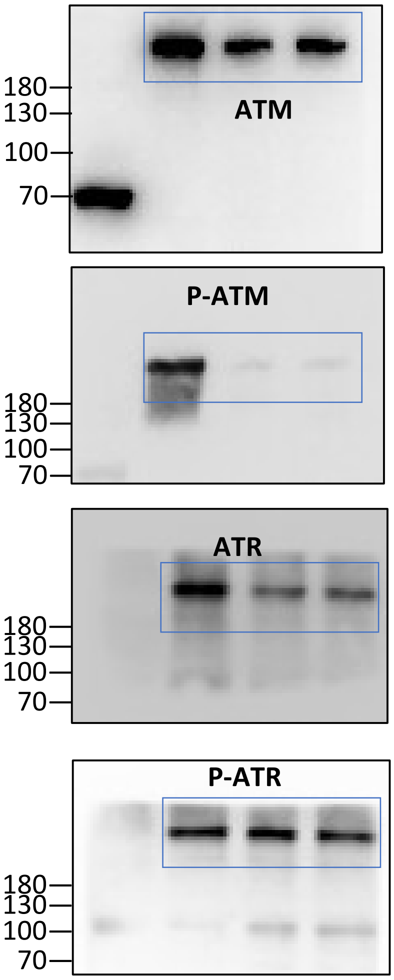

Figure 7D

A2780 cells with CDDP treatment

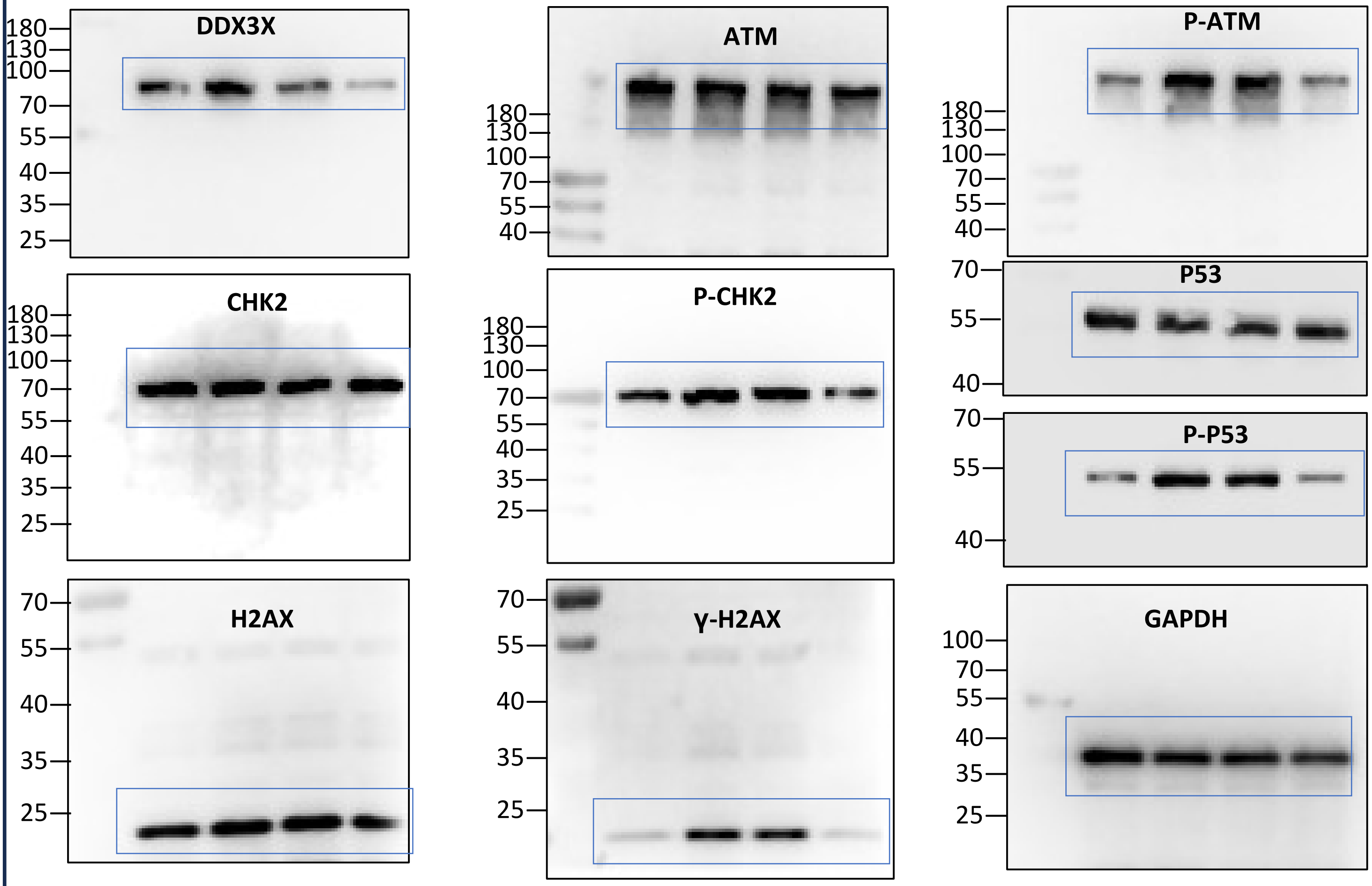

A2780 cells with H_2O_2 treatment

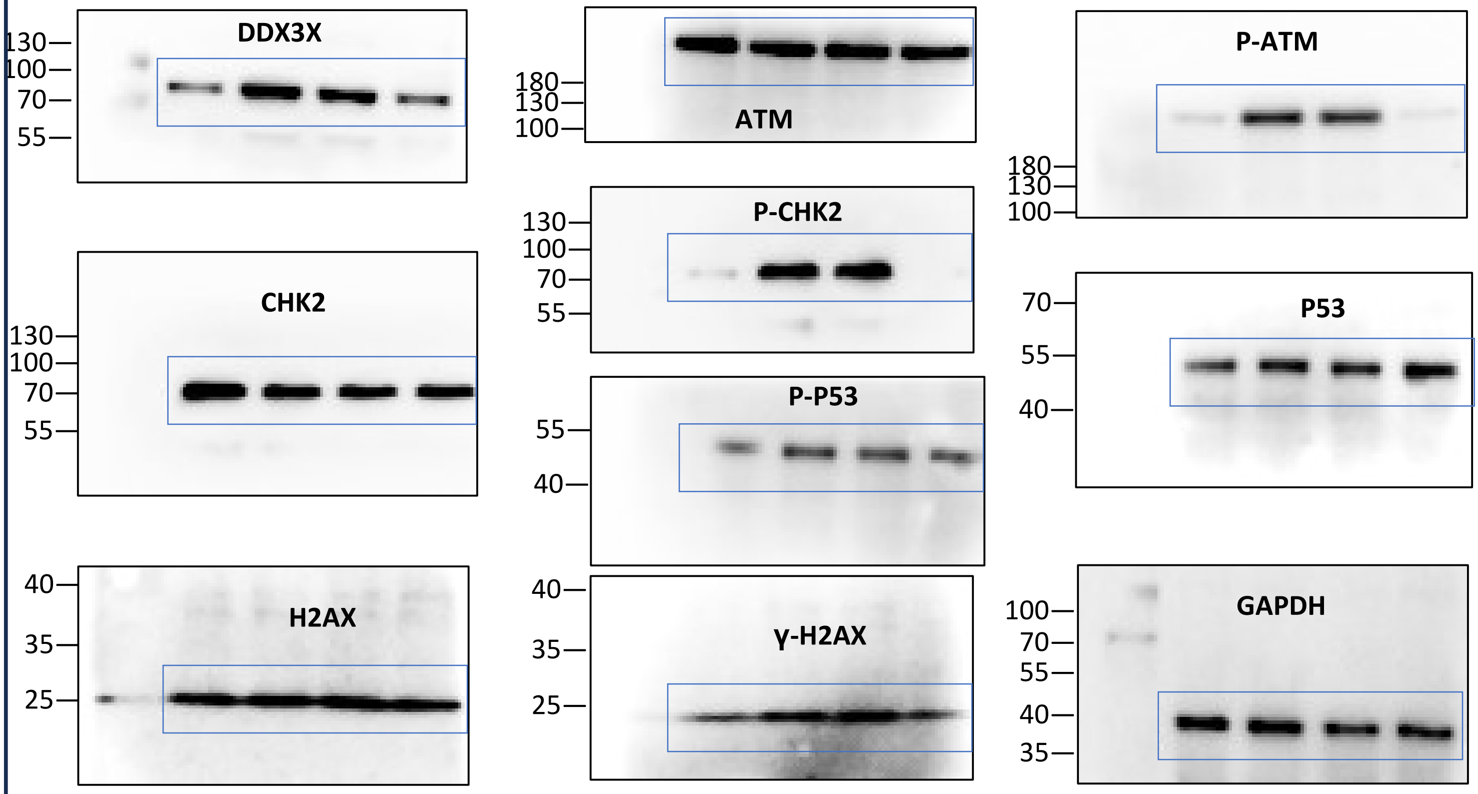

Figure 7E

Hey A8 cells with CDDP treatment

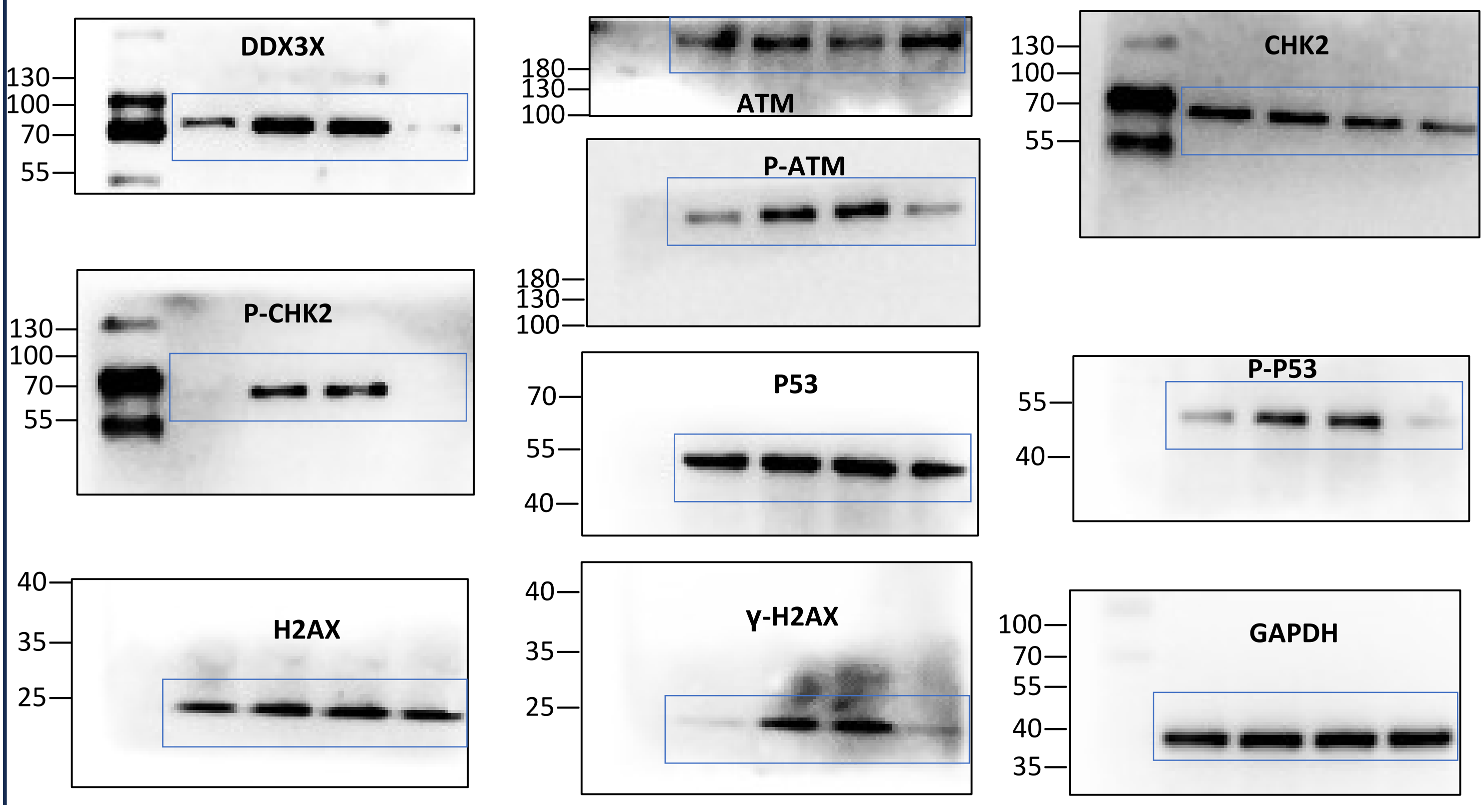

Hey A8 cells with H_2O_2 treatment

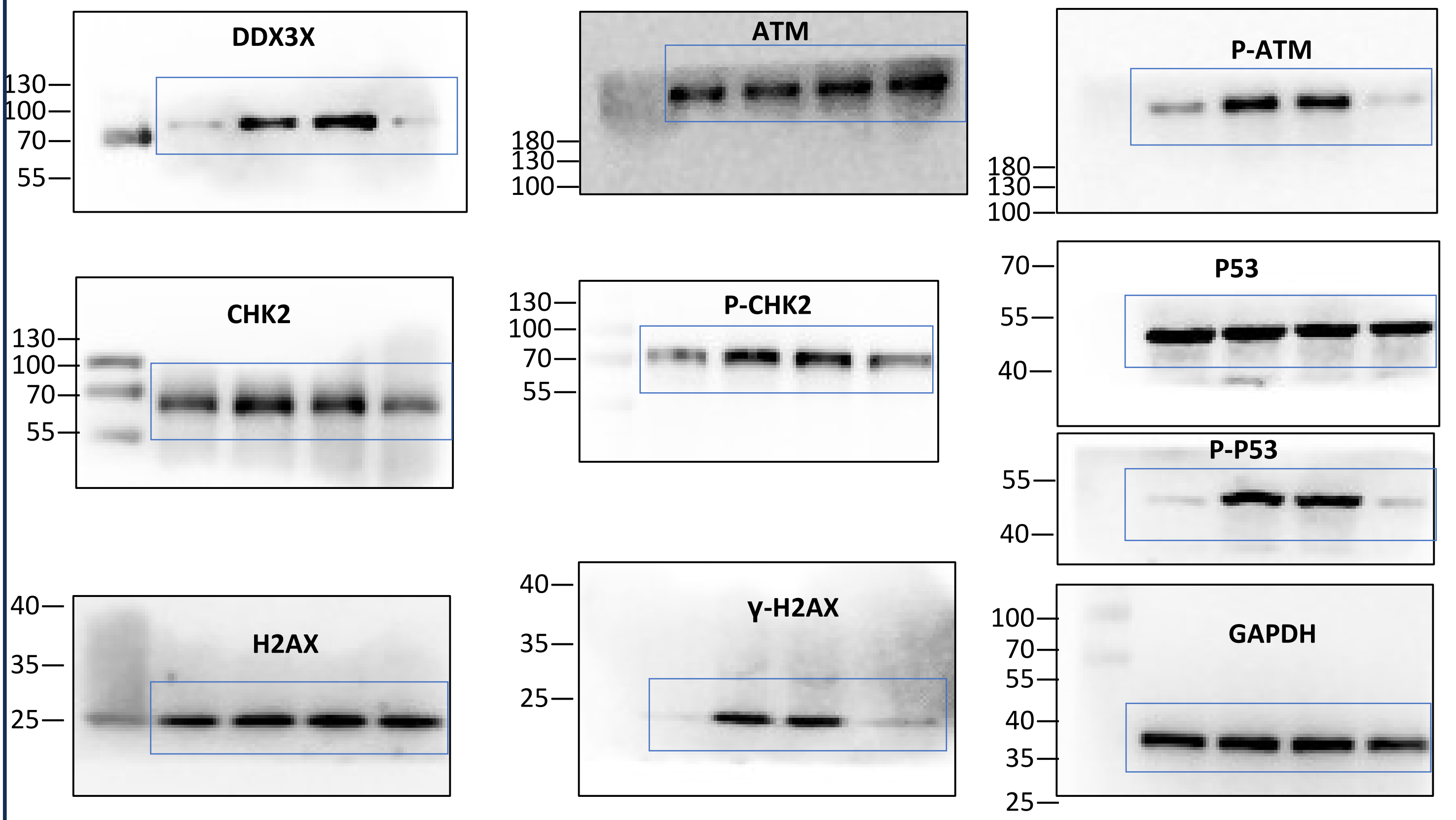

Figure 7F

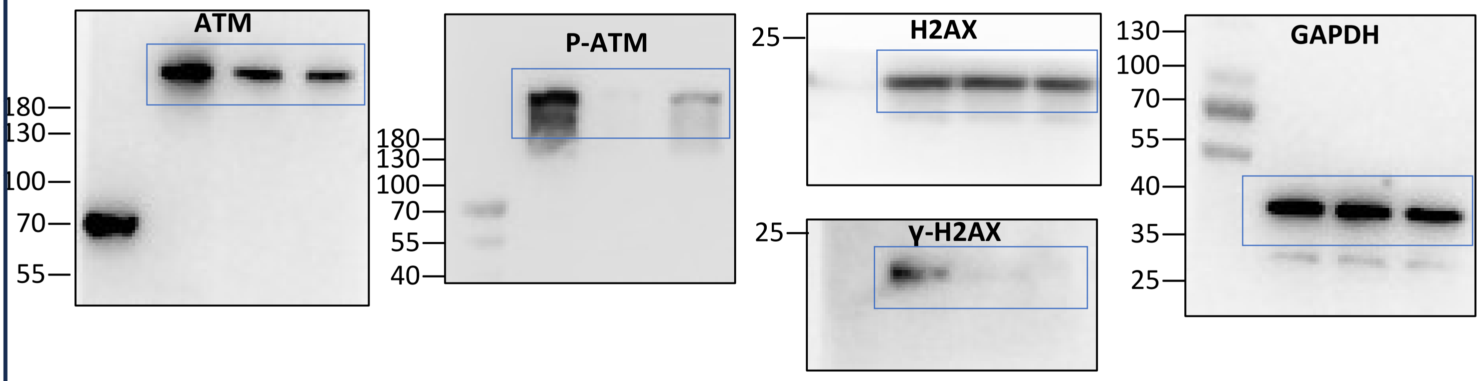

Figure 7J

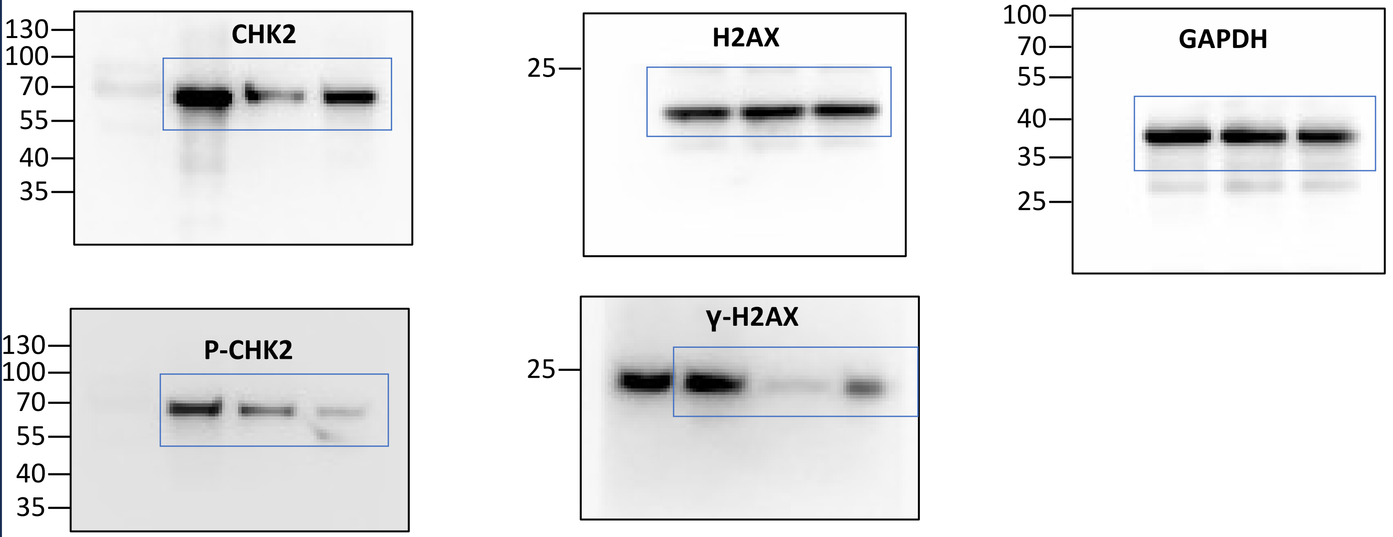

Figure S2B

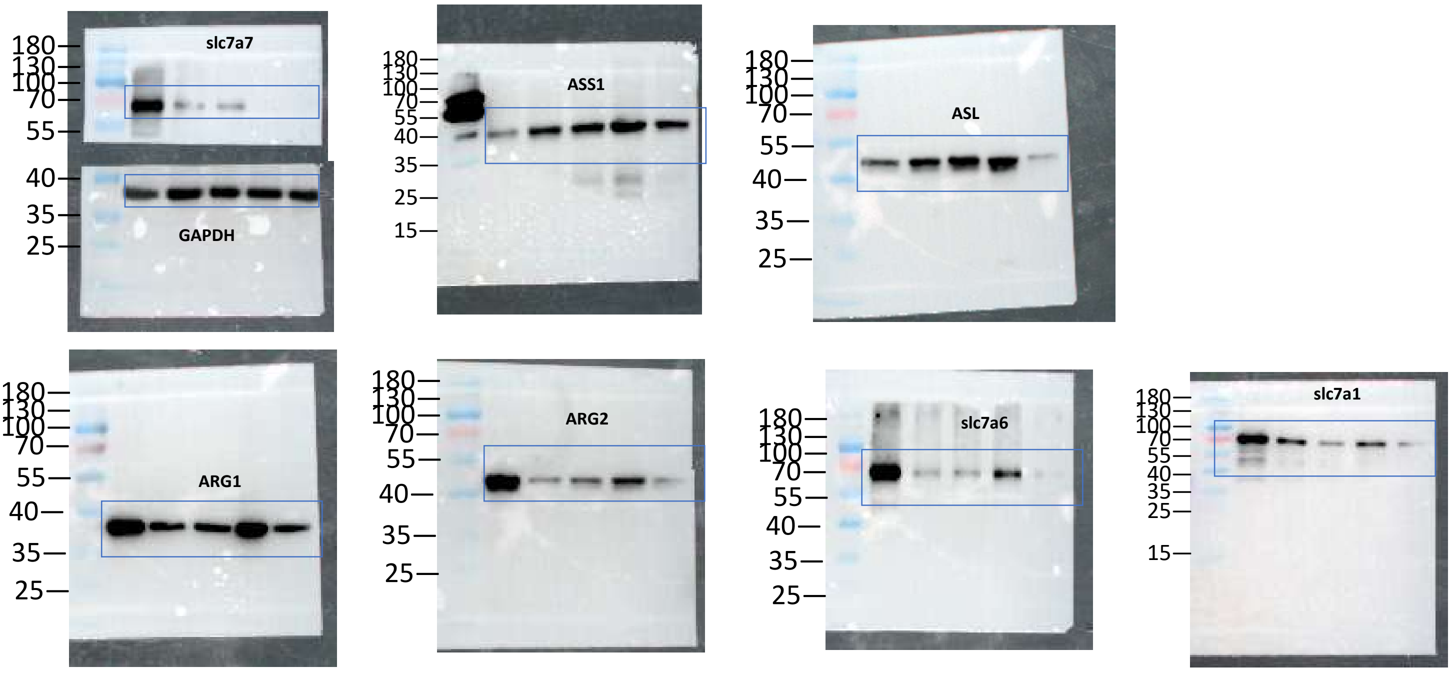

Figure S2C

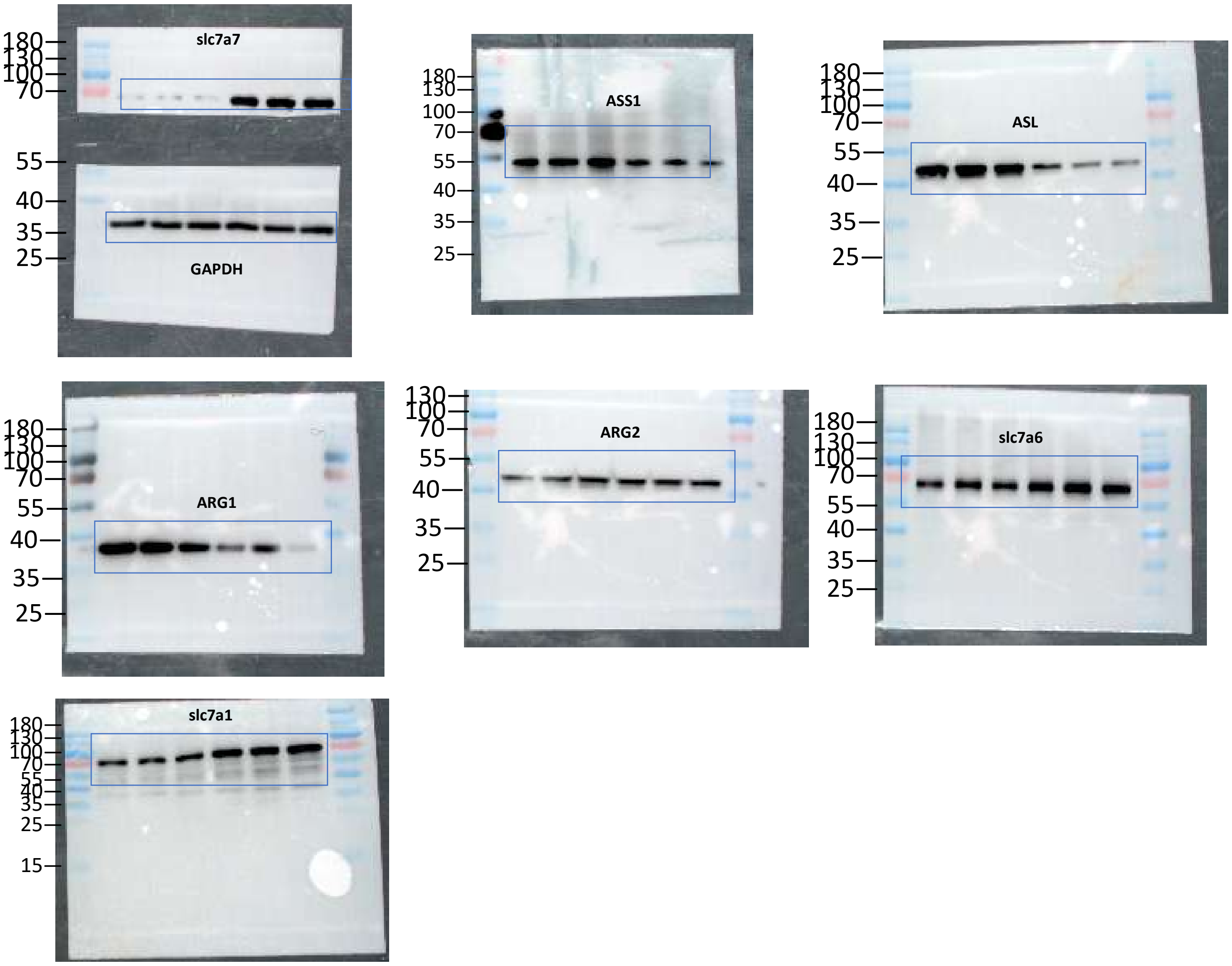

Figure S4A

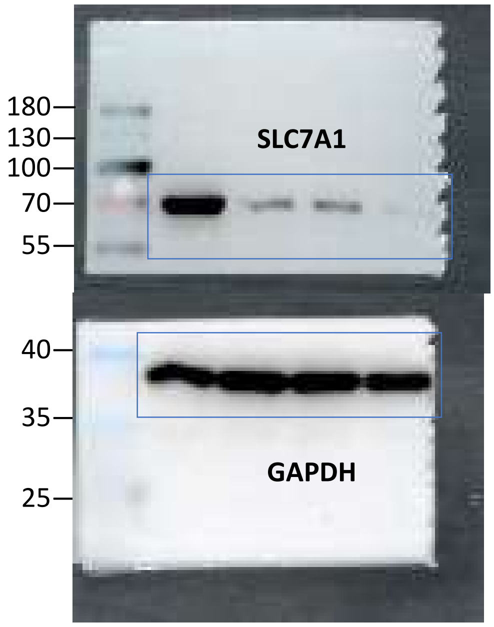

Figure S5E

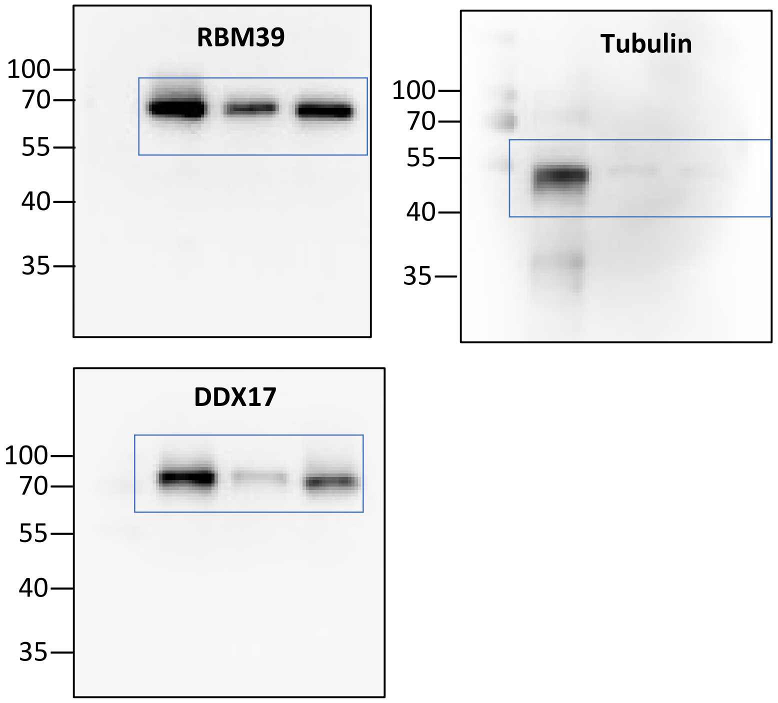

Figure S5H

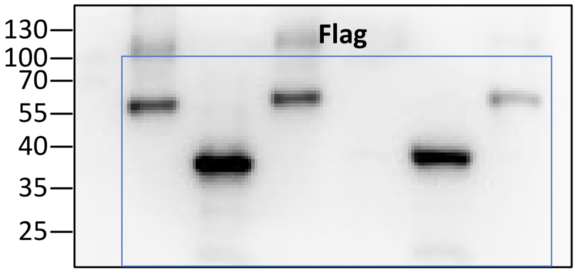

Figure S5I

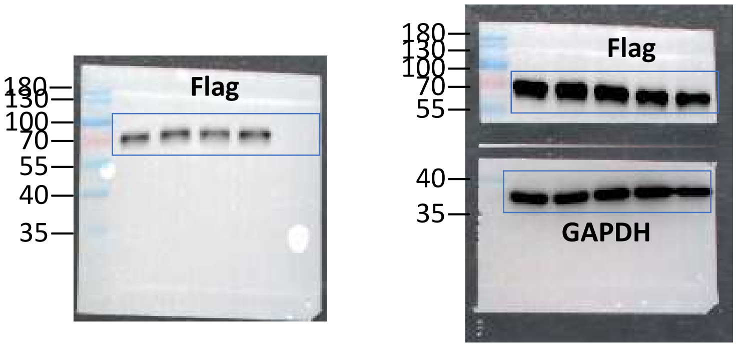

Figure S6A

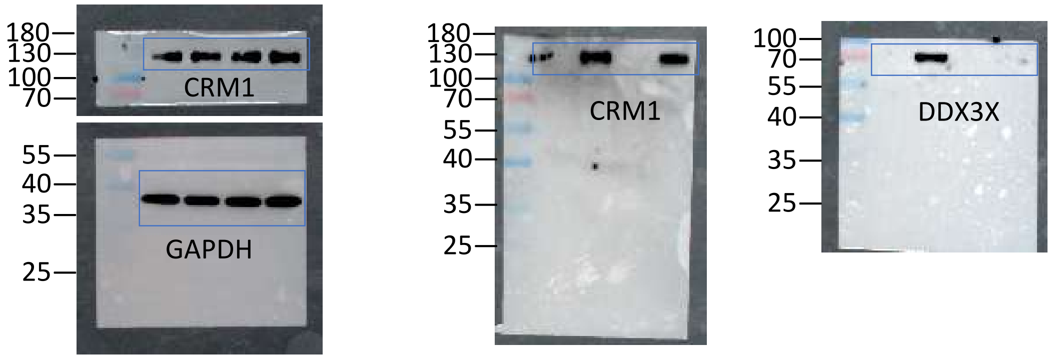

Figure S6E

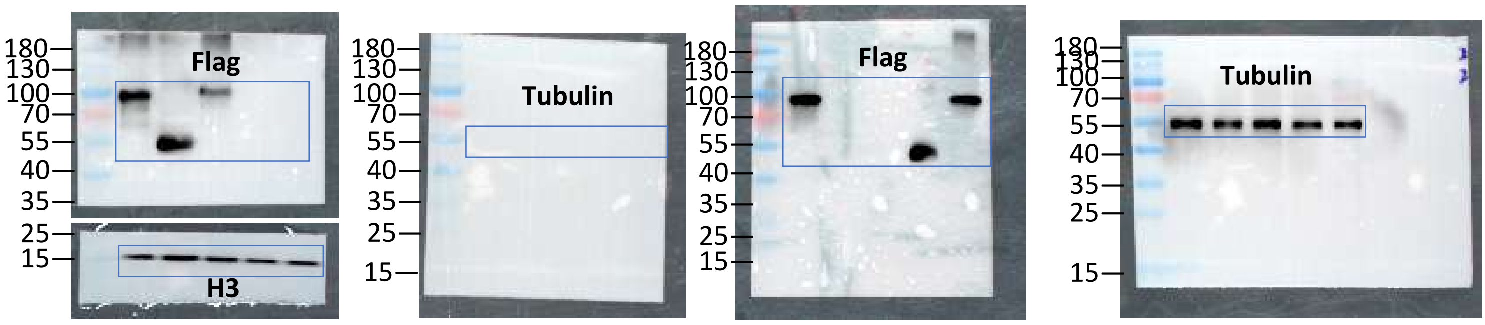

Figure S5A

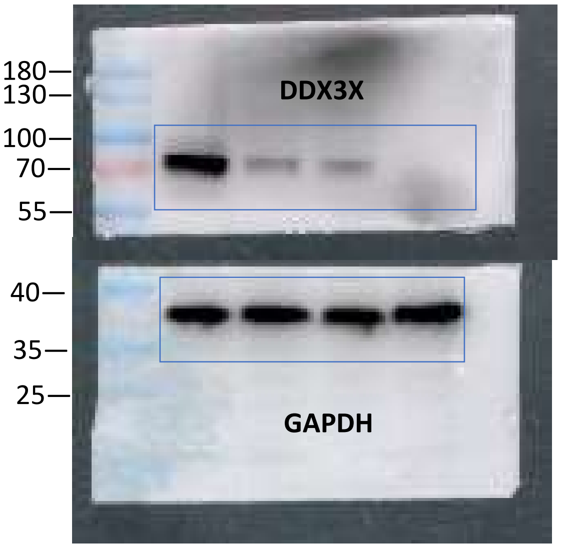

Figure S6C

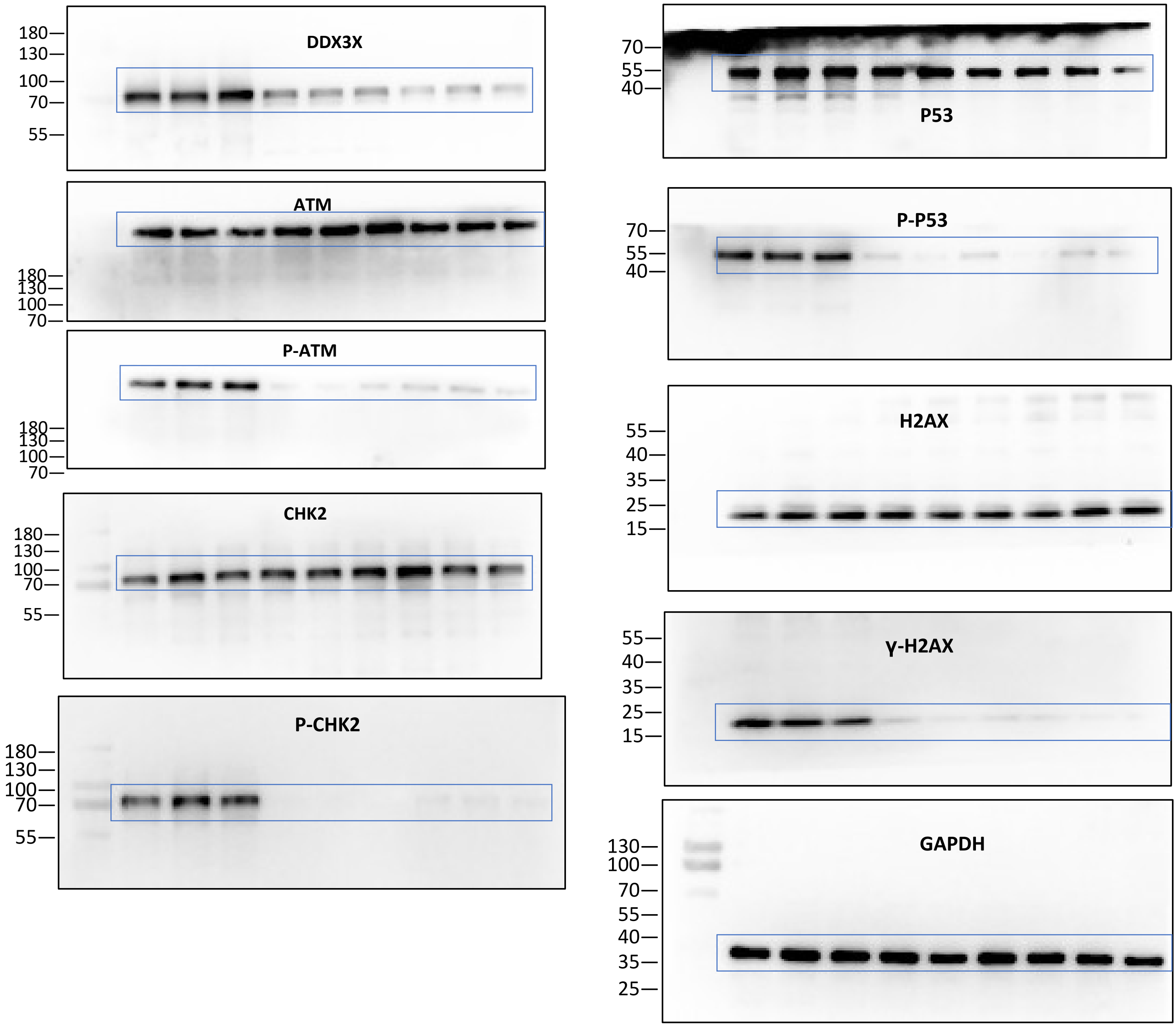

Supplement: Supplementary file 2 — Supplemental data - western blot [file 41419_2026_8606_MOESM2_ESM.pdf]
